# Supplementary material for: RORγ is a targetable master regulator of cholesterol biosynthesis in a cancer subtype
Source: Nat Commun. 2019 Oct 11;10:4621. doi: 10.1038/s41467-019-12529-3 (PMC6789042; doi:10.1038/s41467-019-12529-3)
Supplement: Supplementary file 1 — Supplementary Information [file 41467_2019_12529_MOESM1_ESM.pdf]

Supplementary Information

RORγ is a targetable master regulator of cholesterol biosynthesis in a cancer subtype

Cai et al.

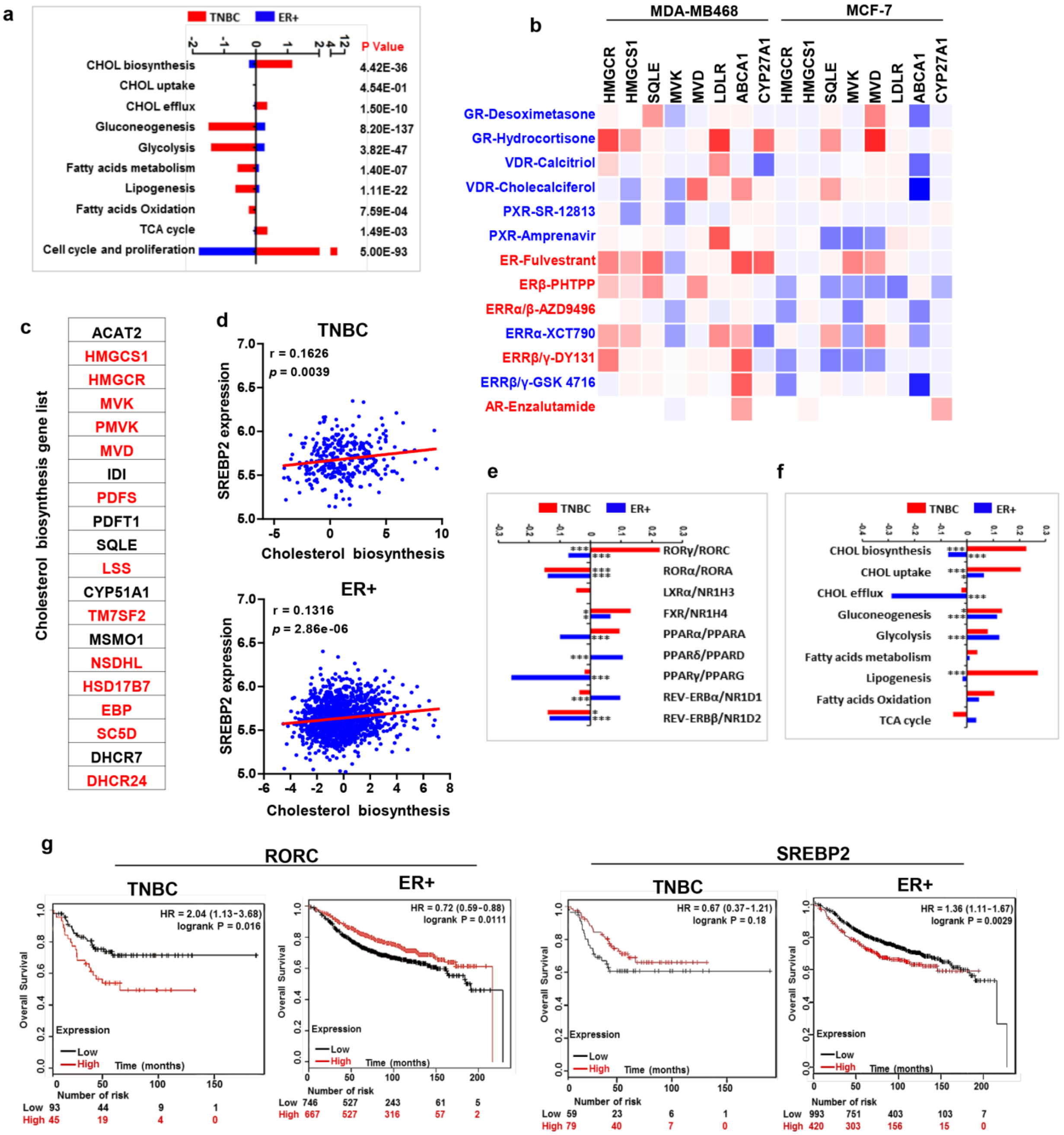

a
TNBC
ER+
P Value
CHOL biosynthesis 4.42E-36
CHOL uptake 4.54E-01
CHOL efflux 1.50E-10
Gluconeogenesis 8.20E-137
Glycolysis 3.82E-47
Fatty acids metabolism 1.40E-07
Lipogenesis 1.11E-22
Fatty acids Oxidation 7.59E-04
TCA cycle 1.49E-03
Cell cycle and proliferation 5.00E-93
b
MDA-MB468
MCF-7
HMGCR HMGCS1 SQLE MVK MVD LDLR ABCA1 CYP27A1
GR-Desoximetasone
GR-Hydrocortisone
VDR-Calcitriol
VDR-Cholecalciferol
PXR-SR-12813
PXR-Amprenavir
ER-Fulvestrant
ERβ-PHTPP
ERRα/β-AZD9496
ERRα-XCT790
ERRβ/γ-DY131
ERRβ/γ-GSK 4716
AR-Enzalutamide
c
Cholesterol biosynthesis gene list
ACAT2
HMGCS1
HMGCR
MVK
PMVK
MVD
IDI
PDFS
PDFT1
SQLE
LSS
CYP51A1
TM7SF2
MSMO1
NSDHL
HSD17B7
EBP
SC5D
DHCR7
DHCR24
d
TNBC
r = 0.1626
p = 0.0039
SREBP2 expression
Cholesterol biosynthesis
ER+
r = 0.1316
p = 2.86e-06
e
RORγ/RORC
RORα/RORA
LXRα/NR1H3
FXR/NR1H4
PPARα/PPARA
PPARδ/PPARD
PPARγ/PPARG
REV-ERBα/NR1D1
REV-ERBβ/NR1D2
f
CHOL biosynthesis
CHOL uptake
CHOL efflux
Gluconeogenesis
Glycolysis
Fatty acids metabolism
Lipogenesis
Fatty acids Oxidation
TCA cycle
g
RORC
SREBP2
TNBC
HR = 2.04 (1.13-3.68)
logrank P = 0.016
ER+
HR = 0.72 (0.59-0.88)
logrank P = 0.0111
TNBC
HR = 0.67 (0.37-1.21)
logrank P = 0.18
ER+
HR = 1.36 (1.11-1.67)
logrank P = 0.0029
Overall Survival
Expression
Low
High
Time (months)
Number of risk
Low 93 44 9 1
High 45 19 4 0
Low 746 527 243 61 5
High 667 527 316 57 2
Low 59 23 6 1
High 79 40 7 0
Low 993 751 403 103 7
High 420 303 156 15 0

Supplementary Fig. 1 RORγ is a putative novel player in control of cholesterol homeostasis and is highly associated with clinical outcome in TNBC.

a Differences in activity scores of indicated pathway gene expression between TNBC and ER+ tumors were analyzed using gene expression datasets from METABRIC consortium. CHOL, cholesterol. Student's *t*-test. **b** Heat map display of fold changes (in log2) of cholesterol homeostasis gene expression in MDA-MB468 and MCF-7 cells treated for 48h by agonists (blue) or antagonists (red) of indicated NRs. The concentration was 2.5 μM for each compound. The expression of indicated genes was analyzed by qRT-PCR. The experiments were repeated three times. **c** Genes involved in cholesterol-biosynthesis were used for correlation analysis. The ones that drive the correlation with RORC expression were labelled in red. The Pearson correlation metric was computed by using the 'cor' function in R. **d** Scatter plots showing correlation of transcript expression between SREBP2 and cholesterol-biosynthesis pathway in TNBC (top) and in ER+ (bottom) tumors. The METABRIC dataset was used. **e** Correlation co-efficiency of transcript expression between cholesterol-biosynthesis pathway and indicated NRs in TNBC and in ER+ tumors. The METABRIC dataset was used. **f** Correlation co-efficiency of transcript expression between RORC and indicated pathways in TNBC and in ER+ tumors. The METABRIC dataset was used. **g** K-M plot of the overall survival determined by RORC (left) or SREBP2 (right) mRNA expression at an optimal threshold for the patients from TNBC and ER + breast cancer. The datasets were analyzed using kmplot.com/analysis/.

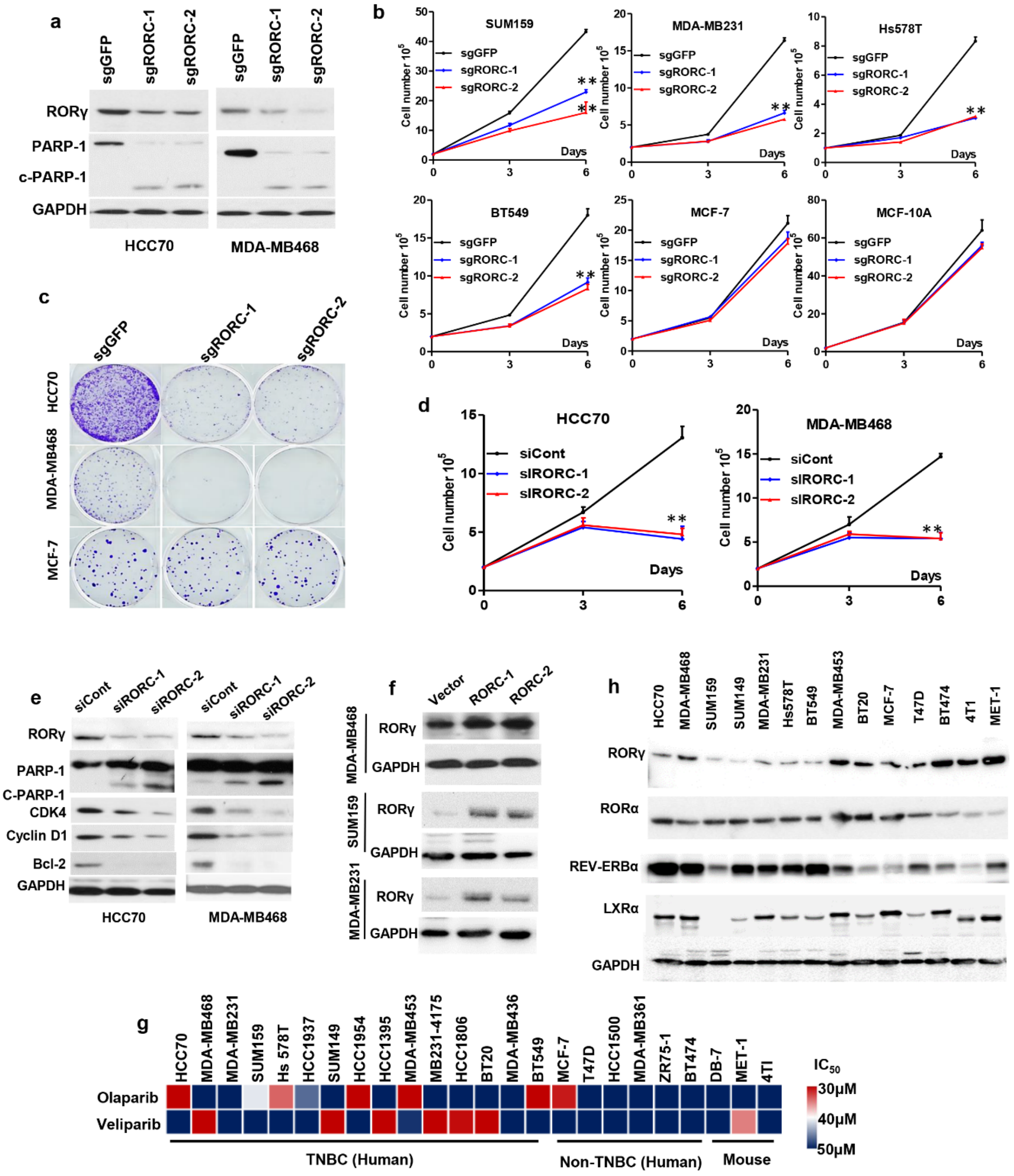
a
sgGFP
sgRORC-1
sgRORC-2
RORγ
PARP-1
c-PARP-1
GAPDH
HCC70
MDA-MB468
b
SUM159
MDA-MB231
Hs578T
BT549
MCF-7
MCF-10A
Cell number 10^5
Days
c
HCC70
MDA-MB468
MCF-7
d
siCont
sIRORC-1
sIRORC-2
e
siCont
siRORC-1
siRORC-2
C-PARP-1
CDK4
Cyclin D1
Bcl-2
f
Vector
RORC-1
RORC-2
SUM159
MDA-MB231
h
SUM149
MDA-MB453
BT20
T47D
BT474
4T1
MET-1
RORα
REV-ERBα
LXRα
g
Olaparib
Veliparib
HCC1937
HCC1954
HCC1395
MB231-4175
HCC1806
MDA-MB436
HCC1500
MDA-MB361
ZR75-1
DB-7
4TI
TNBC (Human)
Non-TNBC (Human)
Mouse
IC50
30μM
40μM
50μM

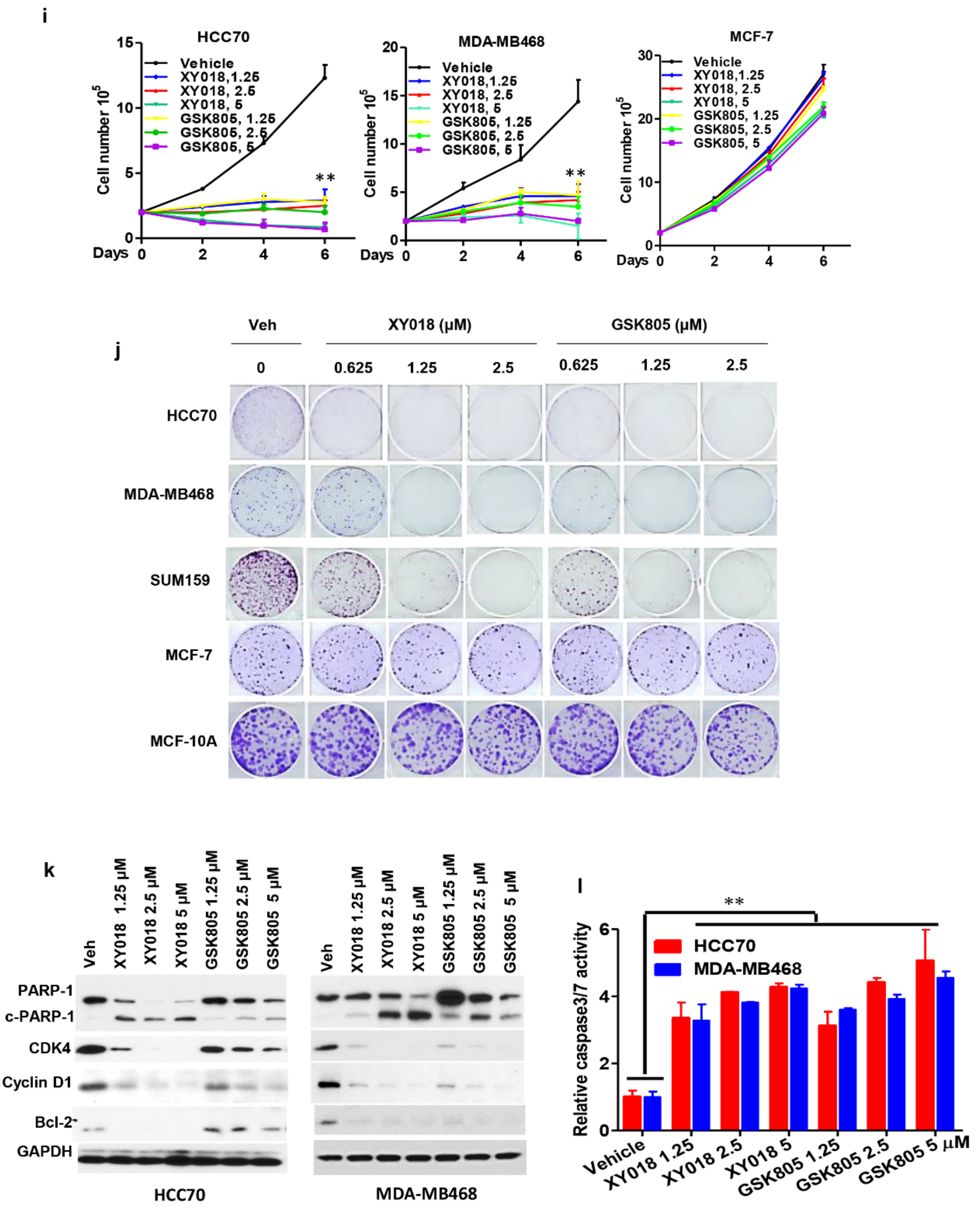
i
HCC70
MDA-MB468
MCF-7
Vehicle
XY018,1.25
XY018, 2.5
XY018, 5
GSK805, 1.25
GSK805, 2.5
GSK805, 5
Cell number 10^5
Days
**
j
Veh
XY018 (μM)
GSK805 (μM)
0
0.625
1.25
2.5
HCC70
MDA-MB468
SUM159
MCF-7
MCF-10A
k
Veh
XY018 1.25 μM
XY018 2.5 μM
XY018 5 μM
GSK805 1.25 μM
GSK805 2.5 μM
GSK805 5 μM
PARP-1
c-PARP-1
CDK4
Cyclin D1
Bcl-2
GAPDH
HCC70
MDA-MB468
l
HCC70
MDA-MB468
Relative caspase3/7 activity
Vehicle
XY018 1.25
XY018 2.5
XY018 5
GSK805 1.25
GSK805 2.5
GSK805 5 μM
**

Supplementary Fig. 2 RORγ is required for TNBC cell survival.

a TNBC cells were infected with lentiviruses expressing control sgRNA against GFP or two sgRNAs against RORC, and Cas9. Three and six days later, RORγ and PARP-1 protein expression was analyzed by immunoblotting. **b** TNBC cells were infected as in (a). Three and six days later, viable cell numbers were counted. **c** TNBC cells were infected as in (a). Fourteen days later, images of colony formation were taken. n = 3. **d** TNBC cells were transfected with control siRNA (siNeal) or two different siRNAs against RORC. Three and six days later, viable cell numbers were counted. **e.** HCC70 cells were transfected as in (d). Three days later, cells were harvested for western blotting analysis of indicated proteins. **f** MDA-MB468, SUM159 and MDA-MB231 cells were infected by RORγ overexpression or control lentivirus. RORγ protein expression was analyzed by immunoblotting. **g** Heat map presentation of IC_{50} PARP-1 inhibitors Olaparib and Veliparib in indicated cell lines treated for 4 days. **h** Immunoblotting analysis of proteins of RORγ, RORα, REV-ERBα and LXRα in TNBC and non-TNBC cell lines. Shown are representative blots. **i** HCC70, MDA-MB468 and MCF-7 cells were treated as indicated for 2, 4 and 6 days. **j** Representative images of colony formation of indicated cells treated with vehicle (DMSO) or the indicated concentrations of XY018 and GSK805 for 14 days. n = 3. **k** TNBC cells were treated with indicated concentrations of XY018 and GSK805. Three days later, cells were harvested for western blotting analysis of indicated proteins. n = 3. **l** Caspase 3/7 activities were measured in TNBC cells treated by indicated the concentrations of XY018 and GSK805 for 3 days. Source data are provided as a Source Data file. Data are showed as mean ± s. d. n = 3. Student's *t*-test. $^{**}p < 0.01$.

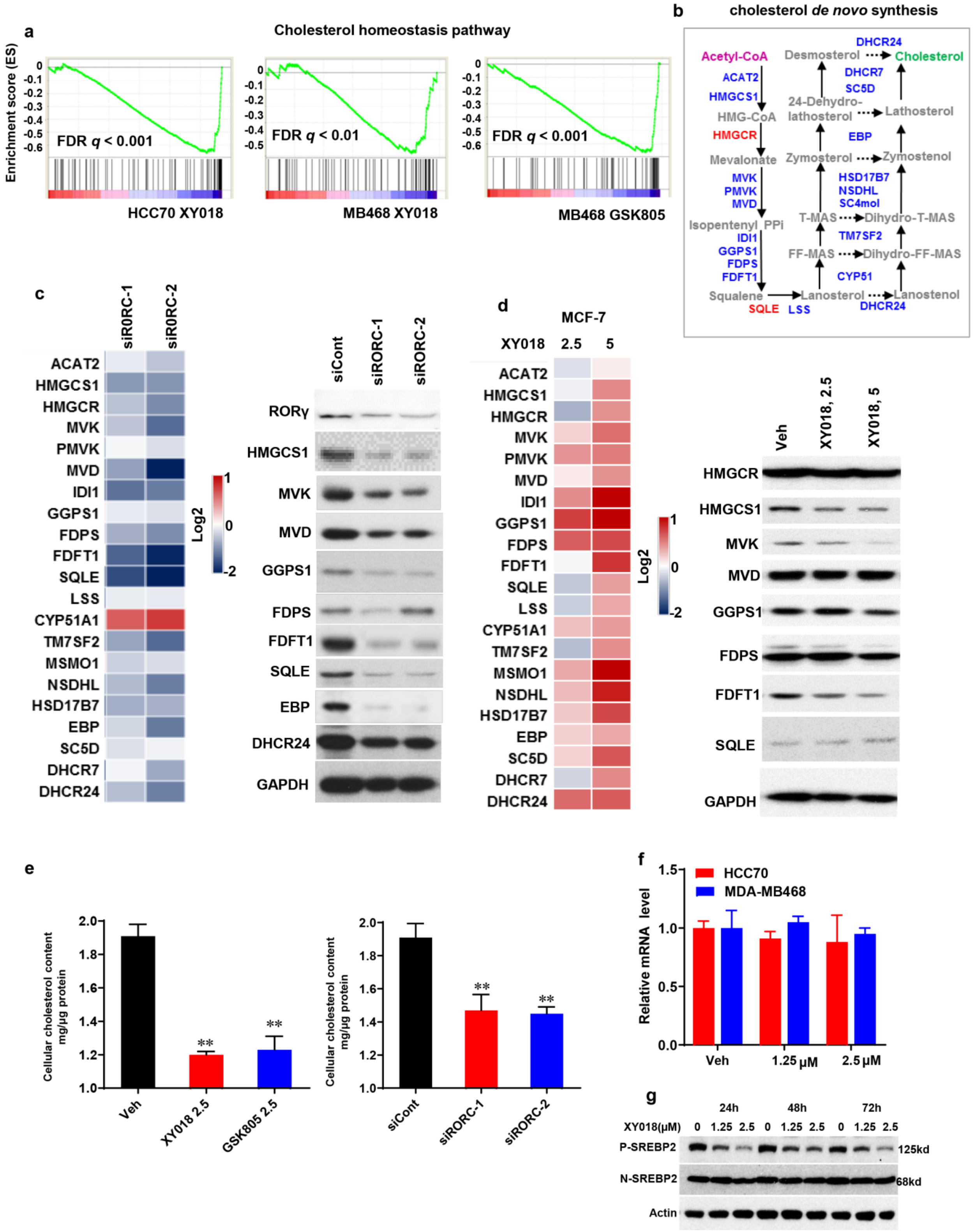

a
Cholesterol homeostasis pathway
Enrichment score (ES)
FDR q < 0.001
HCC70 XY018
FDR q < 0.01
MB468 XY018
FDR q < 0.001
MB468 GSK805
b
cholesterol de novo synthesis
Acetyl-CoA
ACAT2
HMGCS1
HMG-CoA
HMGCR
Mevalonate
MVK
PMVK
MVD
Isopentenyl PPi
IDI1
GGPS1
FDPS
FDFT1
Squalene
SQLE
LSS
Lanosterol
Lanostenol
DHCR24
CYP51
FF-MAS
Dihydro-FF-MAS
TM7SF2
T-MAS
Dihydro-T-MAS
HSD17B7
NSDHL
SC4mol
Zymosterol
Zymostenol
EBP
24-Dehydro-lathosterol
Lathosterol
DHCR7
SC5D
Desmosterol
Cholesterol
c
siRORC-1
siRORC-2
Log2
siCont
RORγ
GAPDH
d
MCF-7
XY018 2.5 5
Veh
XY018, 2.5
XY018, 5
e
Cellular cholesterol content mg/µg protein
Veh
XY018 2.5
GSK805 2.5
siCont
siRORC-1
siRORC-2
f
HCC70
MDA-MB468
Relative mRNA level
Veh
1.25 µM
2.5 µM
g
24h 48h 72h
XY018(µM) 0 1.25 2.5 0 1.25 2.5 0 1.25 2.5
P-SREBP2 125kd
N-SREBP2 68kd
Actin

Supplementary Fig. 3 RORγ antagonists decrease cholesterol biosynthesis in TNBC not in ER+ cells.

a GSEA plots depicting the enrichment of genes down-regulated (1.5 fold) in cholesterol homeostasis pathway in HCC70 cells treated by 2.5 µM XY018 (left), or in MDA-MB468 cells treated by 2.5 µM XY018 (middle) or 2.5 µM GSK805 (right). FDR, false discovery rate. **b** Graphic scheme of cholesterol-biosynthesis pathway from acetyl-CoA. The core genes involved in cholesterol biosynthesis were highlighted in blue, and two rate-limiting enzymes HMGCR and SQLE were highlighted in red. **c** Heat map and representative plots of core cholesterol-biosynthesis gene mRNA and protein expression analyzed by qRT-PCR and immunoblotting in MDA-MB468 cells transfected with control siRNA (siNeal) or two different siRNAs against RORC for 2 or 3 days respectively. Data are showed as mean ± s. d. n = 3. **d** Heat map and representative plots of core cholesterol-biosynthesis gene mRNA and protein expression analyzed by qRT-PCR and immunoblotting in MCF-7 cells treated by indicated the concentrations of XY018. Data are showed as mean ± s. d. n = 3. **e** Total cellular cholesterol contents in MDA-MB468 cells treated by indicated RORγ antagonists (left) or transfected with siRORC (right) for 3 days were analyzed after organic extraction and normalization to protein concentrations. n = 3, Data are showed as mean ± s.d. Student's t-test. ** $p < 0.01$. **f** qRT-PCR analysis of SREBP2 gene expression in the HCC70 and MDA-MB468 cells treated with indicated concentrations of XY018. Data are showed as mean ± s. d, n = 3. **g** Protein contents of pre-mature SREBP2 (P-SREBP2) and nuclear SREBP2 (N-SREBP2) were analyzed by immunoblotting in HCC70 cells treated by indicated concentrations of XY018. n = 3, the experiments were repeated three times. Source data are provided as a Source Data file.

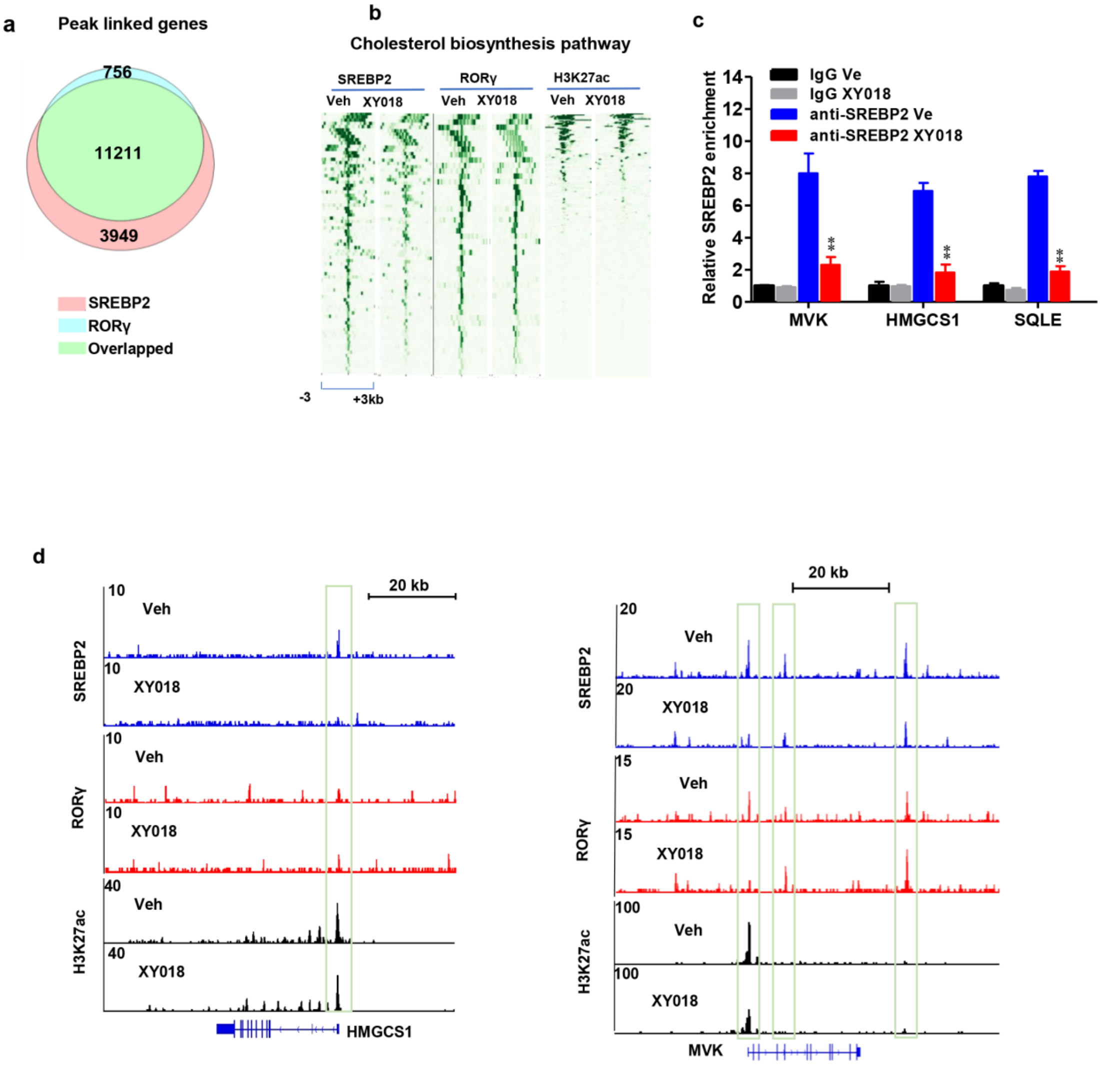
a
Peak linked genes
756
11211
3949
SREBP2
RORγ
Overlapped
b
Cholesterol biosynthesis pathway
SREBP2
RORγ
H3K27ac
Veh
XY018
-3
+3kb
c
Relative SREBP2 enrichment
IgG Ve
IgG XY018
anti-SREBP2 Ve
anti-SREBP2 XY018
MVK
HMGCS1
SQLE
d
20 kb
SREBP2
RORγ
H3K27ac
HMGCS1
MVK

e

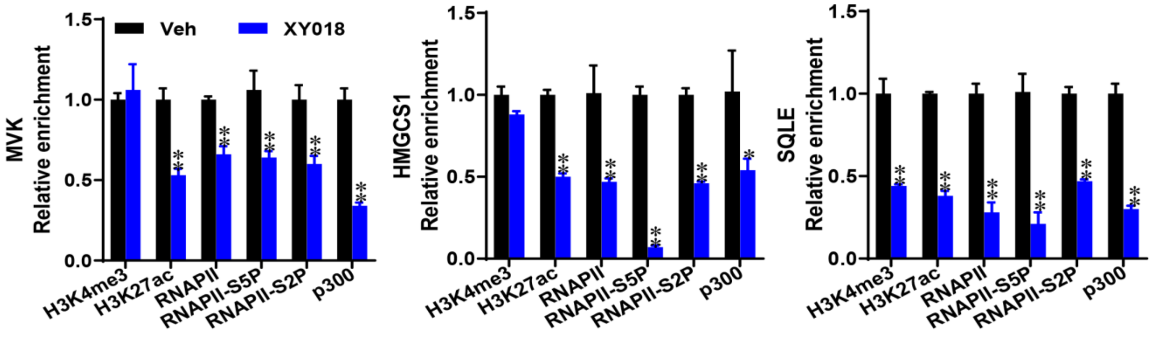

Veh
XY018
MVK
Relative enrichment
HMGCS1
Relative enrichment
SQLE
Relative enrichment
H3K4me3
H3K27ac
RNAPII
RNAPII-S5P
RNAPII-S2P
p300

f

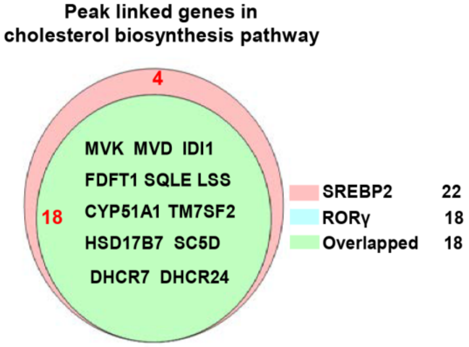

Peak linked genes in
cholesterol biosynthesis pathway
4
18
MVK MVD IDI1
FDFT1 SQLE LSS
CYP51A1 TM7SF2
HSD17B7 SC5D
DHCR7 DHCR24
SREBP2 22
RORγ 18
Overlapped 18

g

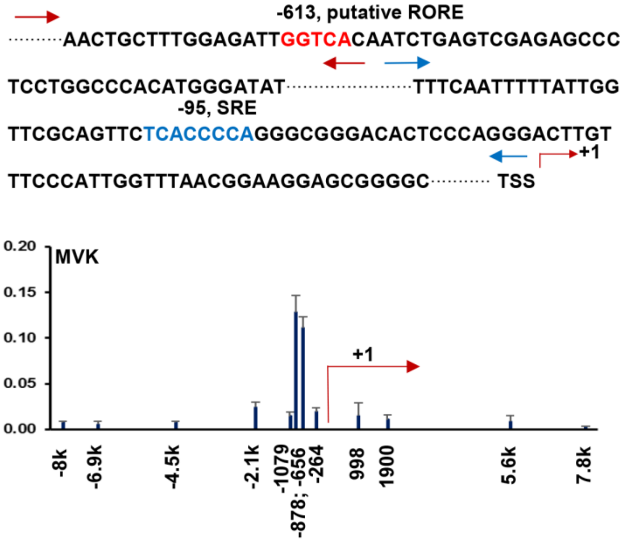

-613, putative RORE
AACTGCTTTGGAGATTGGTCACAATCTGAGTCGAGAGCCC
TCCTGGCCCACATGGGATAT TTTCAATTTTTATTGG
-95, SRE
TTCGCAGTTCTCACCCCAGGGCGGGACACTCCCAGGGACTTGT
+1
TTCCCATTGGTTTAACGGAAGGAGCGGGGC TSS
MVK
Input %
+1
-8k
-6.9k
-4.5k
-2.1k
-1079
-878; -656
-264
998
1900
5.6k
7.8k

h

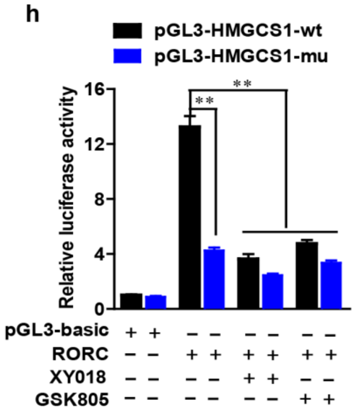

pGL3-HMGCS1-wt
pGL3-HMGCS1-mu
Relative luciferase activity
pGL3-basic
RORC
XY018
GSK805

Supplementary Fig. 4 RORγ antagonists reduce the recruitments of SREBP2, RORγ and cofactors on the promoters of cholesterol-biosynthesis gene in TNBC.

a Venn diagram of the number of genes that displayed ChIP-seq peaks for both RORγ and SREBP2 in HCC70 cells. RORγ or SREBP2 binding sites were assigned to the nearest gene, further annotation information includes whether a peak is in the TSS (transcription start site, from -1kb to +100bp), TTS (transcription termination site, from -100 bp to +1kb), Exon (Coding), 5' UTR Exon, 3' UTR Exon, Intronic, or Intergenic. **b** Heat maps of ChIP-seq signal intensity of SREBP2, RORγ or H3K27ac binding within +/- 3-kb windows around the center of peak regions on genes involved in cholesterol-biosynthesis pathway in HCC70 cells treated by 2.5 μM XY018 or vehicle for 24 hr. **c** ChIP-qPCR analysis of SREBP2 binding on the promoters of cholesterol-biosynthesis gene *HMGCS1*, *MVK* and *SQLE* in HCC70 cells treated by 2.5 μM XY018 for 24 hr. Data are showed as mean ± s. d, n = 3. Student's *t*-test. ** $p < 0.01$. **d** ChIP-seq signal visualization of SREBP2(top), RORγ (middle) or H3K27ac (bottom) binding at representative cholesterol-biosynthesis genes *HMGCS1*and *MVK* in HCC70 cells treated by 2.5 μM XY018 or vehicle for 24 hr. **e** ChIP-qPCR analysis of H3K27ac, p300, RNA polymerase II (RNAPII) or its phosphorylated forms at serine 5 (RNAPII-S5P) or serine 2 (RNAPII-S2P) binding on the promoters of cholesterol-biosynthesis gene *HMGCS1*, *MVK* and *SQLE* in HCC70 cells treated by 2.5 μM XY018 or vehicle for 24 hr. Data are showed as mean ± s. d, n = 3. Student's *t*-test. ** $p < 0.01$. **f** Venn diagram of genes of cholesterol-biosynthesis pathway that displayed ChIP-seq peaks for both RORγ and SREBP2 in HCC70 cells. Peaked linked gene annotation was performed as indicated in (a). **g** Schematic diagram depicting the putative RORE and SRE sequence and primers used for qPCR on the promoter of *MVK* (top). ChIP-qPCR with HCC70 cells revealed that RORγ bound to the sites containing RORE at the upstream of transcription start site (TSS) of *MVK* (bottom). **h** *HMGCS1* (wild type or RORE mutated) promoter luciferase reporter activity changes by RORγ overexpression or treatment with 2.5 μM RORγ antagonists XY018 or GSK805. Data are showed as mean ± s. d. n = 6. Student's *t*-test. **$p < 0.01$. Source data are provided in a Source Data file.

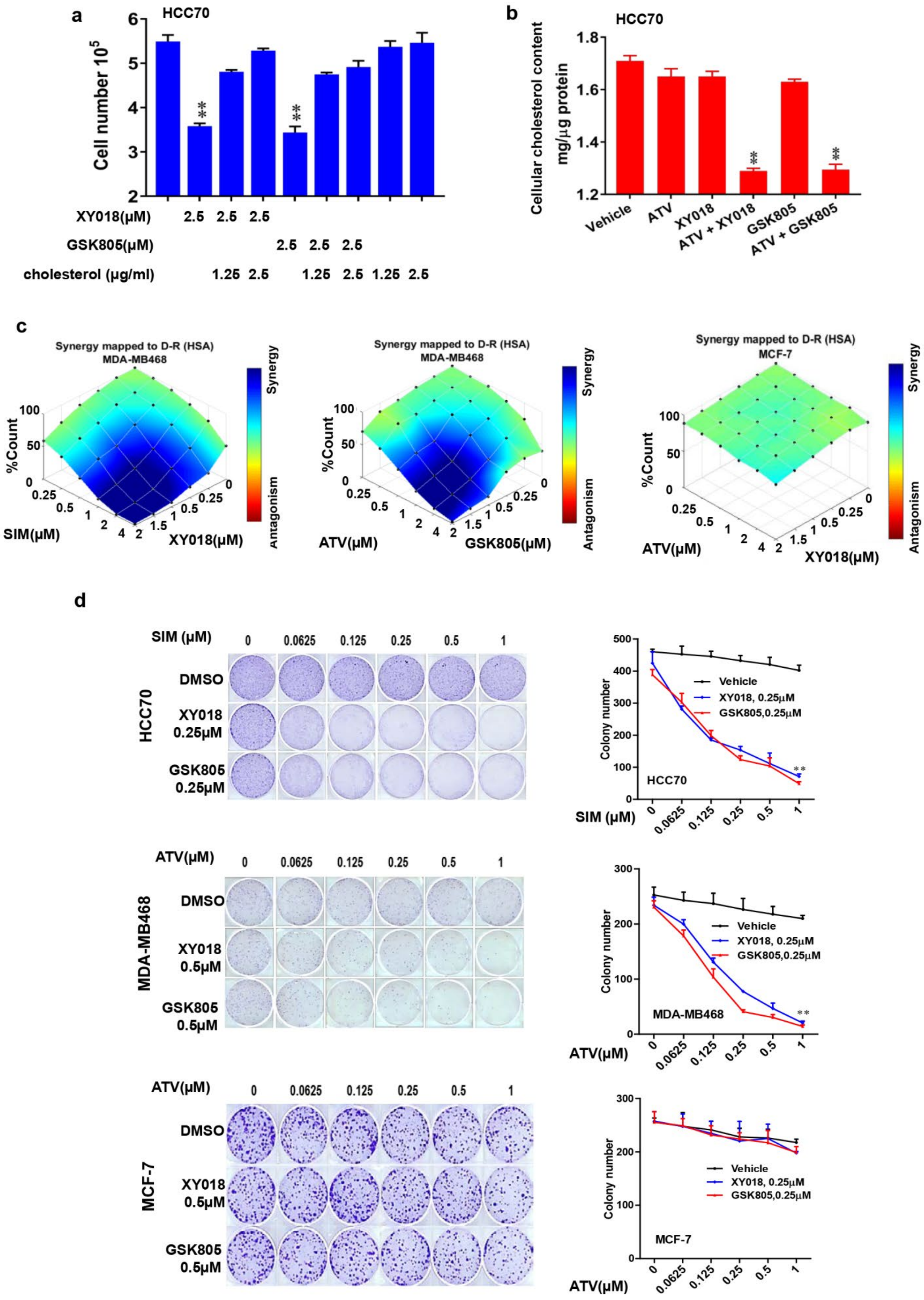

a
HCC70
Cell number 10^5
XY018(μM)
GSK805(μM)
cholesterol (μg/ml)
b
HCC70
Cellular cholesterol content mg/μg protein
Vehicle
ATV
XY018
ATV + XY018
GSK805
ATV + GSK805
c
Synergy mapped to D-R (HSA)
MDA-MB468
MCF-7
%Count
SIM(μM)
XY018(μM)
ATV(μM)
GSK805(μM)
Synergy
Antagonism
d
SIM (μM)
ATV(μM)
HCC70
MDA-MB468
MCF-7
DMSO
XY018 0.25μM
GSK805 0.25μM
XY018 0.5μM
GSK805 0.5μM
Colony number
Vehicle
XY018, 0.25μM
GSK805,0.25μM

e

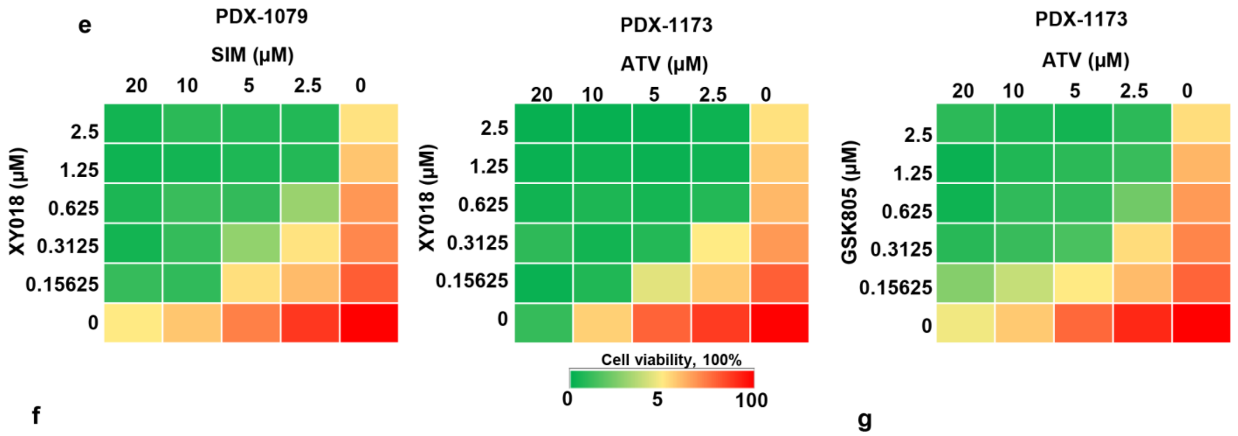

f

Cell line	RORγ inhibitor	IC50 (μM), + or - RORγ inhibitor				
		ATV	SIM	LOV	FLU	ROS
HCC70	–	6.10	3.01	6.58	2.81	55.80
	XY018(0.25 μM)	1.37	0.96	1.79	1.02	17.76
	GSK805(0.25 μM)	1.47	0.92	1.79	1.01	18.48
MDA-MB468	–	10.17	19.26	22.25	12.2	50.00
	XY018(0.25 μM)	2.45	2.21	3.43	2.05	15.34
	GSK805(0.25 μM)	3.54	3.0.2	4.72	2.21	14.63
SUM159	–	10.47	5.00	9.87	11.34	50.00
	XY018(0.25 μM)	4.11	1.60	3.92	3.17	34.33
	GSK805(0.25 μM)	4.52	1.70	4.02	3.34	35.50

g

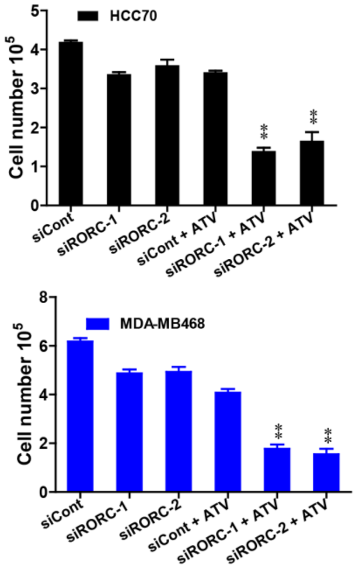

h

Description	LogP
Cholesterol biosynthesis	-8.19
Cholesterol metabolism	-7.92
Sterol biosynthesis	-7.89
Sterol metabolism	-7.29
Steroid metabolism	-5.80
Behavior	-5.70
Steroid biosynthesis	-5.24
Isoprenoid biosynthesis	-4.40

i

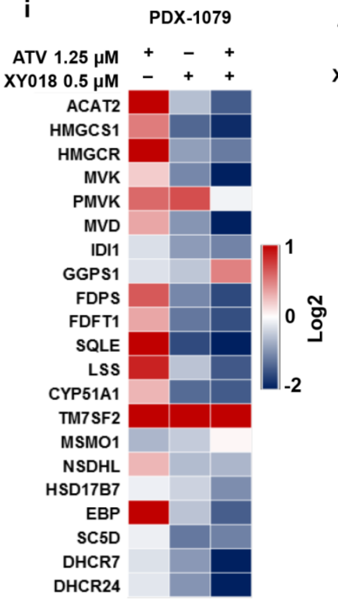

j

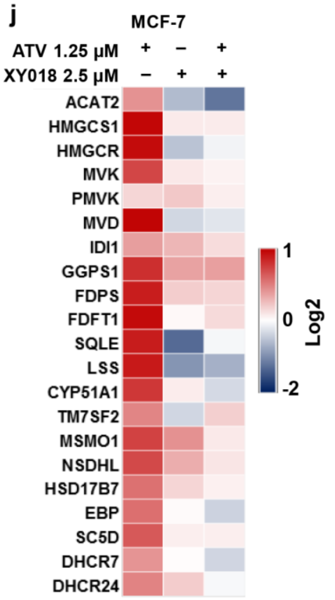

Supplementary Fig. 5 RORγ antagonists in combination with statins synergistically inhibit cell growth and down-regulate cholesterol-biosynthesis gene expression in TNBC, but not in ER+ cells.

a Exogenous cholesterol supply for 24 hr rescued HCC70 cell death caused by treatment with RORγ antagonists for 24 hr. Data are showed as mean ± s. d. n = 3. Student's *t*-test. $^{**}p < 0.01$. **b** Total cellular cholesterol contents in HCC70 cells treated by indicated RORγ antagonists (1.25 μM) or ATV (1.25 μM) for 2 days were analyzed after organic extraction. n = 3, data are showed as mean ± s.d. Student's *t*-test. ** $p < 0.01$. **c** MB468 or MCF-7 cells were treated with RORγ antagonists (XY018 or GSK805) or statins (ATV or SIM) as indicated for 2 days, and cell numbers were counted. For the combinatorial index, blue indicates synergy while red indicates antagonism between drugs. n = 3. The experiments were repeated three times. **d** HCC70, MB468 or MCF-7 cells were treated by indicated concentrations of XY018 or GSK805 alone, or in combination with ATV or SIM for 2 weeks. Representative images of colony formation were taken (left) and colonies were counted (right). Data are showed as mean ± s. d. n = 3. Student's *t*-test. $^{**}p < 0.01$. **e** Organoids derived from PDX-1079 or PDX-1173 were treated with RORγ antagonists or statins, or their combination as indicated for 3 days. The viability was measured by CellTiter-Glo. n = 3, the experiments were repeated three times. **f** IC_{50} of the TNBC cells treated with RORγ antagonist GSK805 or XY018 alone, or in combination with statins as indicated for 4 days. **g** TNBC cells were transfected with control siRNA or siRORC for 24 hr and then treated with 2.5 μM ATV or vehicle (DMSO) for 3 days. Viable cell numbers were counted. Data are showed as mean ± s. d. n = 3. $^{**}p < 0.01$. **h** Gene ontology analysis of 700 up-regulated genes (fold > 1.6, Hypergeometric test and Benjamini-Hochberg p-value correction. $p < 0.0001$) in cholesterol-biosynthesis pathway in MDA-MB468 cells treated by 1.25 μM ATV for 24 hr. **i, j** Heat maps display of fold changes (in log2) of cholesterol-biosynthesis pathway gene mRNA analyzed by qRT-PCR in PDX-1079 derived organoids (i) or MCF-7 cells (j) treated as indicated for 24 hr. n = 3. The experiments were repeated three times.

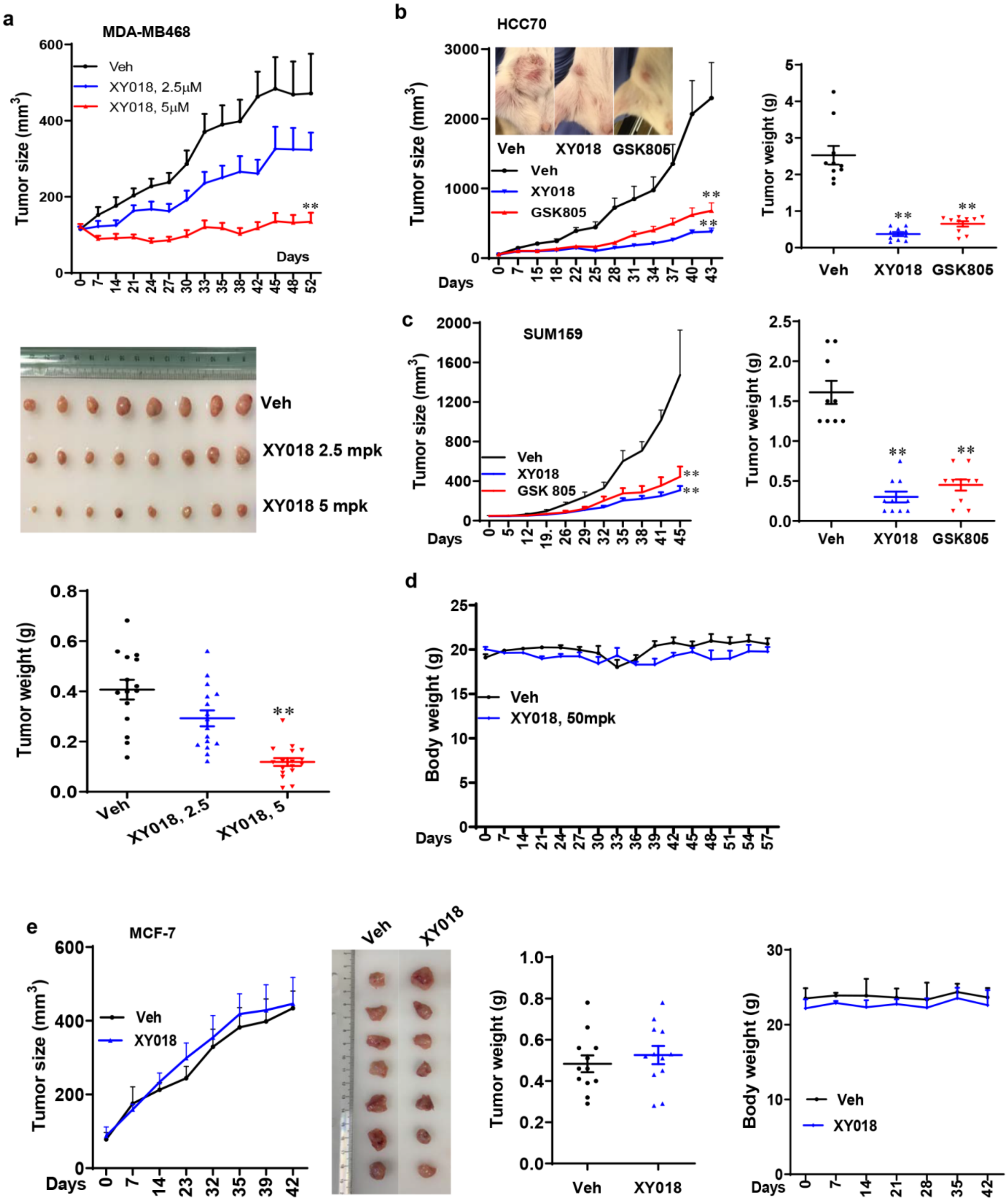
a
MDA-MB468
Veh
XY018, 2.5µM
XY018, 5µM
Tumor size (mm3)
Days
Veh
XY018 2.5 mpk
XY018 5 mpk
Tumor weight (g)
Veh
XY018, 2.5
XY018, 5
b
HCC70
Veh
XY018
GSK805
Tumor size (mm3)
Days
Tumor weight (g)
c
SUM159
Veh
XY018
GSK 805
Tumor size (mm3)
Days
Tumor weight (g)
d
Body weight (g)
Veh
XY018, 50mpk
Days
e
MCF-7
Veh
XY018
Tumor size (mm3)
Days
Tumor weight (g)
Body weight (g)

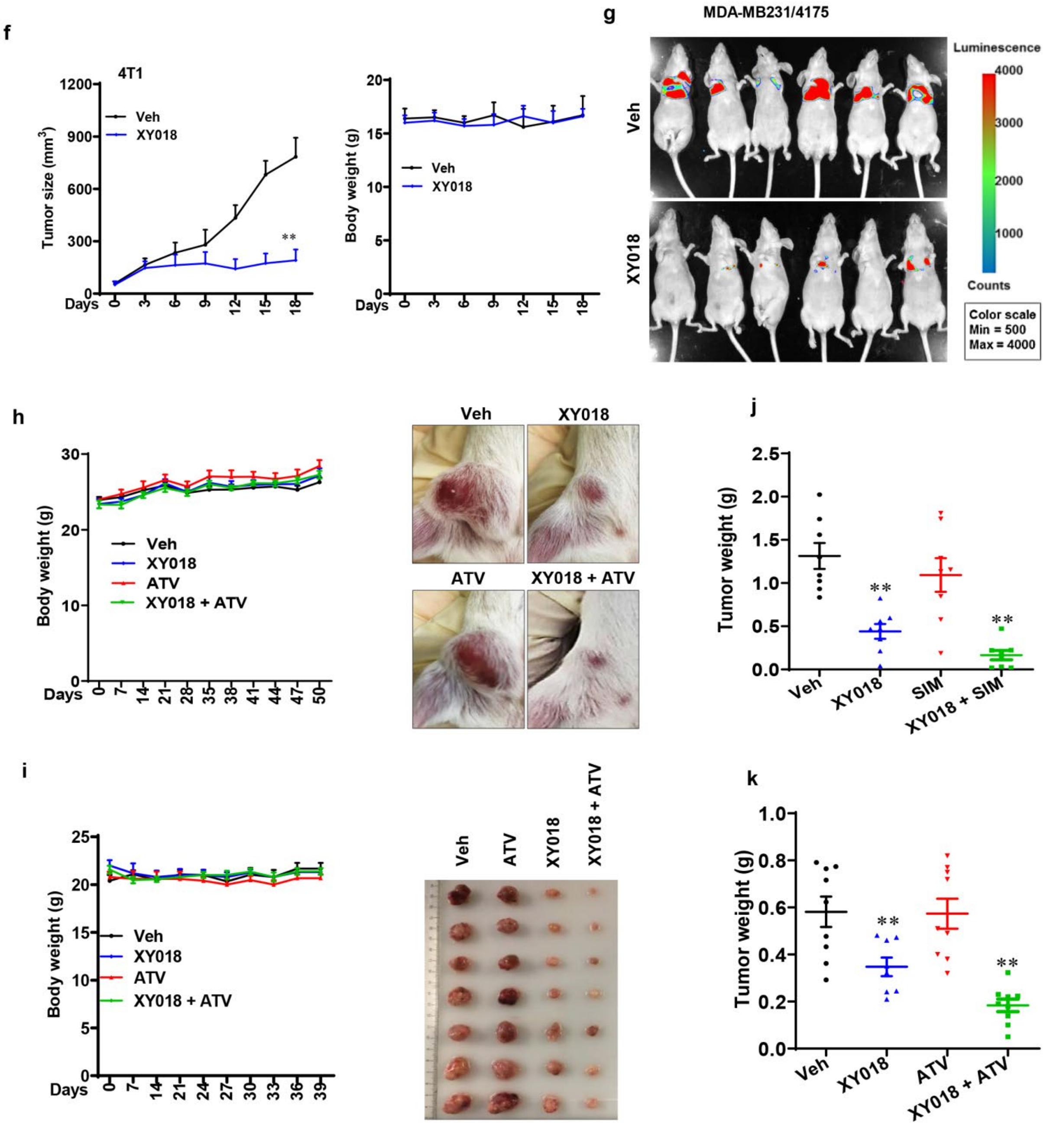
f
4T1
Tumor size (mm3)
Veh
XY018
**
Days
Body weight (g)
g
MDA-MB231/4175
Veh
XY018
Luminescence
Counts
Color scale
Min = 500
Max = 4000
h
Body weight (g)
Veh
XY018
ATV
XY018 + ATV
Days
j
Tumor weight (g)
Veh
XY018
SIM
XY018 + SIM
**
i
Body weight (g)
Veh
XY018
ATV
XY018 + ATV
Days
k
Tumor weight (g)
Veh
XY018
ATV
XY018 + ATV
**

Supplementary Fig. 6 RORγ inhibitors alone or in combination with statins strongly inhibit TNBC tumor growth *in vivo*.

a Mice bearing MDA-MB468 were treated, p.o., 5 times per week, with vehicle or indicated doses of RORγ inhibitor XY018 for 52 days. Tumor volume (top), representative images (middle) and tumor weight (bottom) are shown. n = 7 mice per group. mpk, mg per kg. **b** Mice bearing HCC70 were treated, i.p., 5 times per week, with vehicle or 5 mg/kg XY018 or GSK805 for 43 days. Tumor volume, representative images (left) and tumor weight (right) are shown. n = 7. **c** Mice bearing SUM159 were treated, i.p., 5 times per week, with vehicle or 5 mg/kg XY018 or GSK805 for 45 days. Tumor volume, representative images (left) and tumor weight (right) are shown. n = 7. **d** Body weight of mice bearing PDX-1079 were treated, p.o., 5 times per week, with vehicle or 50 mg/kg RORγ inhibitor XY018 for 57 days. n = 7. **e** Mice bearing MCF-7 were treated, i.p., 5 times per week, with vehicle or 10 mg/kg XY018 for 42 days. Tumor volume, representative images, tumor weight and body weight are shown. n = 7. **f** Mice bearing 4T1 tumors were treated, i.p., 5 times per week, with vehicle for 18 days or 20 mg/kg XY018 for 56 days as shown in Fig. 6b. Tumor volume and body weight are shown. n = 10. **g** Bioluminescence monitoring of MDA-MB231-4175 tumor metastasis in the lung at the indicated days. Nude mice were injected MDA-MB231-4175 cells carrying a luciferase gene via tail vein and treated daily with vehicle or 20 mg/kg XY018 for 4 weeks, n = 6. **h, i** Body weight (left) and representative tumor images (right) of mice bearing PDX-1079 or PDX-1173 treated, 5 times per week, p.o., with vehicle, 20 mg/kg XY018 alone, 15 mg/kg ATV alone, or both XY018 + ATV for indicated days. n = 7 mice per group. **j** PDX-1079 were treated, 5 times per week, p.o., with vehicle, 20 mg/kg XY018 alone, 15mg/kg simvastatin alone (SIM) or both of XY018 + SIM for 47 days. Tumors weight is shown. n = 7. **k** Mice bearing MDA-MB468 were treated, 5 times per week, p.o., with vehicle, 20 mg/kg XY018 alone, 15mg/kg ATV alone or both XY018 + ATV for 32 days. Tumors weight is shown. n = 7.

Throughout, data are showed as mean ± s.e.m.. Student's *t*-test. * $p < 0.05$, ** $p < 0.01$, NS, not significant.

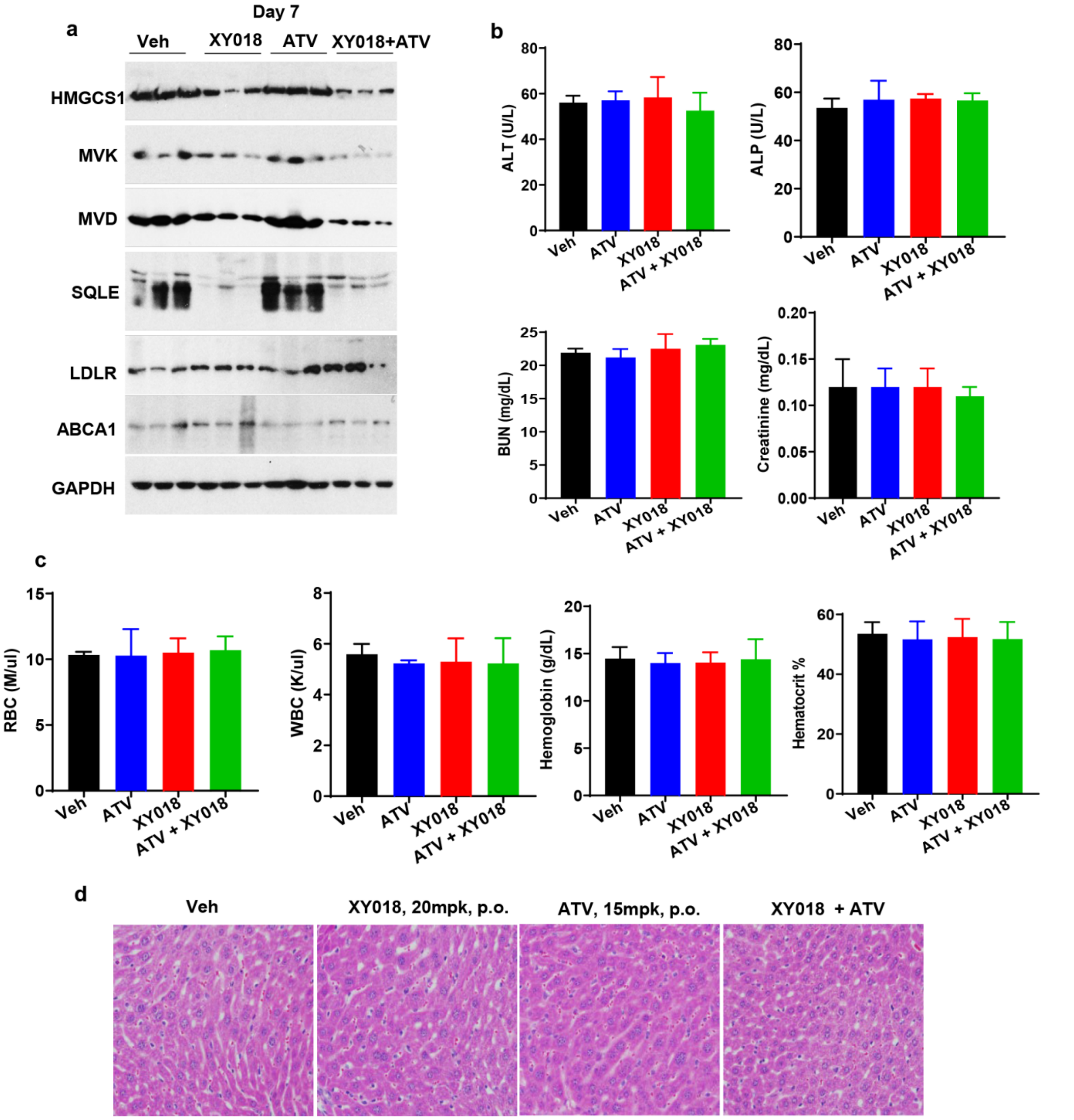

e

Tissue weight	Heart (g)	Liver (g)	Spleen (g)	Lung (g)	Kidney (g)	Ovarian with Oviduct (g)
Veh	0.183 ± 0.012	1.900 ± 0.115	0.267 ± 0.022	0.443 ± 0.012	0.723 ± 0.032	0.430 ± 0.015
ATV,15mpk, p.o.	0.197 ± 0.007	1.953 ± 0.080	0.280 ± 0.015	0.423 ± 0.009	0.743 ± 0.030	0.407 ± 0.009
XY018,20mpk, p.o.	0.197 ± 0.004	1.867 ± 0.012	0.293 ± 0.004	0.430 ± 0.005	0.733 ± 0.008	0.393 ± 0.005
XY018 + ATV	0.190 ± 0.006	1.910 ± 0.035	0.297 ± 0.027	0.440 ± 0.017	0.760 ± 0.035	0.390 ± 0.036

Supplementary Fig. 7 RORγ antagonist reduces protein expression involved in cholesterol-biosynthesis pathway in TNBC tumors without overt toxicity.

a Immunoblotting of proteins involved in cholesterol homeostasis pathway in tumors from HCC70-bearing mice treated with 20 mg/kg XY018 alone, 15mg/kg ATV or XY018 + ATV for 7 days. **b, c** Blood parameters involved in liver and kidney function (b) and complete cell count hematology (c) were measured in MDA-MB468 tumor-bearing mice with indicated treatments for 63 days. n = 7. Student's *t*-test. **d** H&E images of liver from mice as in (b). **e** Weight of indicated tissues from mice as in (b). Data are showed as mean ± s.e.m. n = 7. Student's *t*-test. Source data are provided in a Source Data file.

Supplementary Table 1. Gene list for each of the programs related to Fig. 4b.

Description	Gene list
Circadian entrainment	ADCY1, ADCY2, ADCY3, ADCY4, ADCY5, ADCY7, ADCY8, ADCY9, ADCYAP1R1, CACNA1C, CACNA1D, CACNA1G, CACNA1H, CACNA1I, CALM1, CALM2, CALML3, CALML6, CAMK2B, CAMK2D, CAMK2G, FOS, GNAI1, GNAI2, GNAQ, GNAS, GNB1, GNB2, GNB3, GNB4, GNB5, GNG10, GNG12, GNG13, GNG4, GNG8, GNGT2, GRIA2, GRIA4, GRIN1, GRIN2A, GRIN2B, GRIN2C, GRIN2D, GUCY1A2, ITPR3, KCNJ3, KCNJ9, MAPK1, MAPK3, MTNR1A, MTNR1B, NOS1, NOS1AP, PER1, PER3, PLCB1, PLCB3, PLCB4, PRKACA, PRKACB, PRKACG, PRKCA, PRKCB, PRKCG, PRKG1, PRKG2, RASD1, RYR1, RYR2, RYR3
Steroid hormone biosynthesis	AKR1C2, COMT, CYP11A1, CYP7B1, HSD11B2, HSD17B7, HSD17B8, SRD5A1, SRD5A2, UGT2B4, UGT2B7
Cortisol synthesis and secretion	ADCY1, ADCY2, ADCY3, ADCY4, ADCY5, ADCY7, ADCY8, ADCY9, AGTR1, ATF4, CACNA1C, CACNA1D, CACNA1F, CACNA1G, CACNA1H, CACNA1I, CREB3, CREB3L1, CREB5, CYP11A1, GNA11, GNAQ, GNAS, ITPR2, ITPR3, KCNA4, KCNK2, KCNK3, LDLR, MC2R, NR0B1, NR4A1, NR5A1, PBX1, PDE8B, PLCB1, PLCB3, PLCB4, POMC, PRKACA, PRKACB, PRKACG, SCARB1
Drug metabolism	DPYS, GMPS, GSTA5, GSTM1, GSTM3, GSTM5, GSTO1, GUSB, HPRT1, IMPDH1, MGST2, MPO, NME3, NME4, TK2, UCK1, UCKL1, UGT2B4, UGT2B7, UPP1
Autophagy	AKT1, AKT1S1, AKT2, AKT3, ATG12, ATG16L1, ATG2A, ATG4C, ATG4D, ATG9B, BCL2, BECN2, BNIP3, CFLAR, DAPK1, DAPK2, DEPTOR, EIF2AK3, GABARAPL2, HMGB1, HRAS, IGF1R, IRS1, IRS2, IRS4, KRAS, LAMP1, MAP2K2, MAP3K7, MAPK1, MAPK3, MAPK8, MAPK9, MLST8, MRAS, MTMR3, MTMR4, NRAS, PIK3CA, PIK3CB, PIK3CD, PIK3R2, PPP2CB, PRKAA1, PRKAA2, PRKACA, PRKACB, PRKACG, PRKCD, PRKCQ, RAB33B, RAB7A, RAF1, RB1CC1, RHEB, RPTOR, RRAGA, RRAGC, RRAS, RRAS2, RUBCN, SH3GLB1, STK11, STX17, SUPT20H, TP53INP2, TSC2, ULK1, UVRAG, WIPI2, ZFYVE1

Supplementary Table 2. Primers used for qPCR and ChIP assay

Primers for human qPCR		Primers for mouse qPCR	
ACAT rtF	GCAGGTGTTCCTTCAATGGT	Hmgcs1-F	AGGAACGTGGTATCTGGTCA
ACAT rtR	CACAGCTTTTAGGCCTGACC	Hmgcs1-R	TGTGTTACTATGCACGAGCC
HMGCS1 rtF	AGCTCAGAGAGGACACCCAT	Hmgcr-F	ATGGCTGGGAGCATAGGCGG
HMGCS1 rtR	GGTACTTTCTTGGCAGGGCT	Hmgcr-R	CTGCATCCTGGCCACATGCG
HMGCR rtF	CCCAGCCTACAAGTTGGAAA	Mvk-F	AGGTCCCGCGGAGTACCAAG
HMGCR rtR	GCTCCCATCACCAAGGAGTA	Mvk-R	CTAGCACGCGCTCACACTCC
MVK rtF	GCTCAAGTTCCCAGAGATCG	Mvd-F	TCGAGTGTGATGGGCAGCCA
MVK rtR	ATGGTGCTGGTTCATGTCAA	Mvd-R	CAGAAACCAGCGGGGAACCG
PMVK rtF	CGGAGAGTGTCTGACATCCA	Fdps-F	ACAACCGGGGTTTGACCGTG
PMVK rtR	AAGTTGTCCAGGCCACATTC	Fdps-R	TGTCAGGGCCCGCTGAAGAC
MVD rtF	AGGACAGCAACCAGTTCCAC	Fdft1-F	CCTTGCCCTCAGCAGCCTGG
MVD rtR	GTGTCGTCCAGGGTGAAGAT	Fdft1-R	GCACGCTGCCAGTGGCTACA
IDI1 rtF	TGTTCCCTGCGAAAGGTATC	Sqle-F	CCGTTTACAGCCAGGCGAGC
IDI1 rtR	TGAACCTGTTGCTTGTCGAG	Sqle-R	ACTGATGGACACGGGCCTCT
GGPS rtF	ACACGGTGAAACCCTGTCTC	Idi1-F	GGCCATGACACTCAACCCAGC
GGPS rtR	AGAGGCACTATCTCGGCTCA	Idi1-R	TGGGCCAAACATCCAAGGGCT
FDPS rtF	AGGGCAATGTGGATCTTGTC	Cyp51-F	CCCTCAGACGGTGGCAGGGT
FDPS rtR	GAAAGAACTCCCCCATCTCC	Cyp51-R	GTCCAAGCGCTCTGCCCAGG
FDFT1 rtF	GGTCCCGCTGTTACACAACT	Dhcr7-F	GGGCTGCAAGCCTGGCTCATT
FDFT1 rtR	AAAACTCTGCCATCCCAATG	Dhcr7-R	TGCGAACGTGGACACGGCAT
SQLE rtF	GGCCATCTTTTGTTGGAGAA	Ldlr-F	ACCTGCCGACCTGATGAATTC
SQLE rtR	TTCAGAAGGGAATGGGAGTG	Ldlr-R	GCAGTCATGTTCACGGTCACA
LSS rtF	TTCCTGAGGCTCTCACAGGT	Abca1-F	GGCTCCTCCCTGTTTTTGAA
LSS rtR	CCCTCCATCTGGATTTCTCA	Abca1-R	GAACTGAGGGACGATTCCAC
CYP51 rtF	GCTCAGTTGTTCCCTGCTTC		
CYP51 rtR	AAAATTAGCCAGGCATGGTG		
TM7SF rtF	CGCTTTCATCTTCAGCCTCT		
TM7SF rtR	GCTCTGCCTCCTTCATCAAC		
SC4MOL rtF	ACTCTGTCTCCTTGGCTGGA		
SC4MOL rtR	CATCGTGAAACCCCATCTCT		
NSDHL rtF	CTCAGCCAGTCACTCCTTCC		
NSDHL rtR	CTGCCTGCTTCAAGAAATCC		
HSD17B7 rtF	CGTAGGACTTCCGAAAGCAG		
HSD17B7 rtR	AGACAGCTTCTGCCTTGCTC		
EBP rtF	GCCTCAGCACCTAAGACTGG		
EBP rtR	ATGAACCCACACACTGCAAA		
SC5D rtF	TATCTCTTCCGCCCATGTTC		
SC5D rtR	TGGCTCATTCACCATTTCAA		

DHCR7 rtF	CATTGACATCTGCCATGACC		
DHCR7 rtR	ACAGGTCCTTCTGGTGGTTG		
DHCR4 rtF	TGTTGCCTGAGCTTGATGAC		
DHCR4 rtR	GACCAGGGTACGGCATAGAA		
**Primers for ChIP-qPCR**			
MVK SRE F	GCCATGAGCCAGGCTGCTTGA		
MVK SRE R	GTGTTGCAGCCAAGCCGACAC		
HMGCS1 SRE F	GCCGTATCTCGCAGCTCCGTCA		
HMGCS1 SRE R	GCCGGTAGAGTTGCCCGGCAG		
SQLE SRE F	GCGAGCACACCTCAGTCCAG		
SQLE SRE R	GCTAGGGCGATGTTGCGTGGTGC		
MVK -8K F	GCAAACTGAGATCAGTGCTCTG		
MVK -8K R	GCAGAGCCAGGGTCCTGACCTG		
MVK -6.9K F	GCTCCTCCTCAACTGAGAGACG		
MVK -6.9K R	GCGGGCCTGACTAGGTGAGAT		
MVK -4.5K F	GCCTCAGCCTCCCAAAGTGCTG		
MVK -4.5K R	GCTTTCAGGGTGTTGCTCATGTT		
MVK -2.1K F	GCATGAGCCACCGCGCCTGGTA		
MVK -2.1K R	GCCACAGCCTTCCTCGAAGACA		
MVK -1079 F	GCATGATCATAGCTCACTGCAGC		
MVK -1079 R	GCAATTCTTCATCTCTGAGTAC		
MVK -878 F	GCCAAAATACAGCTCAAAAAG		
MVK -878 R	GCTTTCCTCATATTCCTTGAAAC		
MVK -656 F	GCTGGTGTATCTGGGACCTGGG		
MVK -656 R	GCTGTTCTGACGTCACCATGGC		
MVK -264 F	GCCAAGACGGCTCCCCAGGCCA		
MVK -264 R	GCCCTGGGGTGAGAACTGCGAAC		
MVK +998 F	GGACGGTAACTACCTTTTTTG		
MVK +998 R	GCCATGTACCACGGCATGTTC		
MVK +1.9K F	GCTGGTCTCAAACTCCTGGGCTCA		
MVK +1.9K R	GCCTGCAAAATTTCAGAGGTTCTC		
MVK +5.6K F	GCCATGTTACCTAGGCTGGTCTCA		
MVK +5.6K R	GCCTCTTCTTACGGTTGTCAGGAA		
MVK +7.8K F	GCAGCCCTCCTGACTGTGTGCG		
MVK +7.8K R	GCCATTTGACCTCTCGGGGCTCA		

Supplementary Table 3. Antibodies used in immunoblotting

Antibody	Vendor	Catalog number	Dilution
RORγ	Ebioscience	14-6988-82	1:1000
LXRα	abcam	ab176323	1:1000
RORα	Santa Cruz	sc-518081	1:1000
CDK4	Santa Cruz	sc-260	1:1000
Cyclin D1	NeoMarkers	MS-260-P1	1:1000
BCL-2	Santa Cruz	sc-7382	1:1000
SREBP-2	Cayman	10007663	1:1000
LDLR	Proteintech	10785-1-AP	1:1000
ABCA1	Santa Cruz	SC-58219	1:1000
GAPDH	Cell signaling	#2118	1:3000
HMGCR	Santa Cruz	sc-271595	1:1000
HMGCS1	Santa Cruz	sc-166763	1:500
MVK	Proteintech	12228-1-AP	1:1000
MVD	Santa Cruz	sc-376975	1:1000
GGPS1	Santa Cruz	sc-271680	1:1000
FDPS	Proteintech	16129-1-AP	1:1000
FDFT1	Santa Cruz	SC-271602	1:1000
SQLE	Santa Cruz	sc-271651	1:1000
EBP	Santa Cruz	sc-374267	1:1000
DHCR24	Santa Cruz	sc-398938	1:1000
PARP-1	Cell signaling	#9542	1:1000
REV-ERBα	Cell signaling	#13418	1:1000
