# Supplementary material for: RORγ is a targetable master regulator of cholesterol biosynthesis in a cancer subtype
Source: Nat Commun. 2019 Oct 11;10:4621. doi: 10.1038/s41467-019-12529-3 (PMC6789042; doi:10.1038/s41467-019-12529-3)
Supplement: Supplementary file 3 — Reporting Summary [file 41467_2019_12529_MOESM3_ESM.pdf]

nature research

Corresponding author(s): Hong-wu Chen

Last updated by author(s): 8/6/2019

Reporting Summary

Nature Research wishes to improve the reproducibility of the work that we publish. This form provides structure for consistency and transparency in reporting. For further information on Nature Research policies, see Authors & Referees and the Editorial Policy Checklist.

Statistics

For all statistical analyses, confirm that the following items are present in the figure legend, table legend, main text, or Methods section.

n/a	Confirmed	
☐	☒	The exact sample size (*n*) for each experimental group/condition, given as a discrete number and unit of measurement
☐	☒	A statement on whether measurements were taken from distinct samples or whether the same sample was measured repeatedly
☐	☒	The statistical test(s) used AND whether they are one- or two-sided<br>*Only common tests should be described solely by name; describe more complex techniques in the Methods section.*
☒	☐	A description of all covariates tested
☒	☐	A description of any assumptions or corrections, such as tests of normality and adjustment for multiple comparisons
☐	☒	A full description of the statistical parameters including central tendency (e.g. means) or other basic estimates (e.g. regression coefficient) AND variation (e.g. standard deviation) or associated estimates of uncertainty (e.g. confidence intervals)
☐	☒	For null hypothesis testing, the test statistic (e.g. *F*, *t*, *r*) with confidence intervals, effect sizes, degrees of freedom and *P* value noted<br>*Give P values as exact values whenever suitable.*
☒	☐	For Bayesian analysis, information on the choice of priors and Markov chain Monte Carlo settings
☒	☐	For hierarchical and complex designs, identification of the appropriate level for tests and full reporting of outcomes
☒	☐	Estimates of effect sizes (e.g. Cohen's *d*, Pearson's *r*), indicating how they were calculated

Our web collection on statistics for biologists contains articles on many of the points above.

Software and code

Policy information about availability of computer code

Data collection: None computer code

Data analysis: RNA-seq: The FASTQ-formatted sequence data were analyzed using a standard BWA-Bowtie-Cufflinks workflow. Sequence reads were mapped to GRCh37/hg19 assembly with BWA and Biotie software.
ChIP-seq: Fastq files were processed by the pipeline of AQUAS Transcription Factor and Histone (https://github.com/kundajelab/chipseq_pipeline). Sequencing tags were mapped against the Homo sapiens (human) reference genome (hg19) using BWA 0.7.15. Uniquely mapped tags filtering and deduping were used for peak calling by model-based analysis for ChIP-Seq (MACS; 2.1.0) to identify regions of ChIP-seq enrichment over background.

For manuscripts utilizing custom algorithms or software that are central to the research but not yet described in published literature, software must be made available to editors/reviewers. We strongly encourage code deposition in a community repository (e.g. GitHub). See the Nature Research guidelines for submitting code & software for further information.

Data

Policy information about availability of data

All manuscripts must include a data availability statement. This statement should provide the following information, where applicable:
- Accession codes, unique identifiers, or web links for publicly available datasets
- A list of figures that have associated raw data
- A description of any restrictions on data availability

All data supporting the findings of this study are available within the article and Supplementary Information. RNA-seq and ChIP-seq data generated in this study are deposited in the Gene Expression Omnibus (GEO) database under the accession number GSE131856 (RNA-seq) and GSE126380 (ChIP-seq).

Field-specific reporting

Please select the one below that is the best fit for your research. If you are not sure, read the appropriate sections before making your selection.

☒ Life sciences ☐ Behavioural & social sciences ☐ Ecological, evolutionary & environmental sciences

For a reference copy of the document with all sections, see nature.com/documents/nr-reporting-summary-flat.pdf

Life sciences study design

All studies must disclose on these points even when the disclosure is negative.

Sample size	No statistical method was used to pre-determine sample size.
Data exclusions	No data were excluded from analyses.
Replication	The reproducibility for each analysis was confirmed by three independent experiments.
Randomization	No randomization in this study.
Blinding	No blinding in this study.

Reporting for specific materials, systems and methods

We require information from authors about some types of materials, experimental systems and methods used in many studies. Here, indicate whether each material, system or method listed is relevant to your study. If you are not sure if a list item applies to your research, read the appropriate section before selecting a response.

Materials & experimental systems

n/a	Involved in the study	
☐	☒	Antibodies
☐	☒	Eukaryotic cell lines
☒	☐	Palaeontology
☐	☒	Animals and other organisms
☒	☐	Human research participants
☒	☐	Clinical data

Methods

n/a	Involved in the study	
☐	☒	ChIP-seq
☒	☐	Flow cytometry
☒	☐	MRI-based neuroimaging

Antibodies

Antibodies used	BCL-2(1:1000, sc-7382); FDPS (1:1000, 16129-1-AP); MVK (1:1000, 12228-1-AP), LDLR (1:1000, 10785-1-AP), SREBP-2 (1:1000, Cayman,10007663); RORγ (1:1000,Ebioscience,12-6988-82); CDK4 (1:500, sc-260); PARP-1 (1:1000, Cell signaling #9542), HMGCR (1:1000, sc-271595); HMGCS1 (1:1000, sc-166763); SQLE (1:1000, sc-271651); FDFT1 (1:1000, sc-271602); GGPS1 (1:1000, sc-271680); DHCR24 (1:1000, sc-398938); EBP (1:1000, sc-374267); MVD (1:1000, sc-376975); ABCA1 (1:500, SC-58219); LXRα(1:1000,ab176323); REV-ERBα (1:1000, Cell signaling #13418); RORα(1:1000, sc-sc-518081).
Validation	Validation statement of each antibody noted on the manufacturer website.

Eukaryotic cell lines

Policy information about cell lines

Cell line source(s)	HCC70, HCC1500, HCC1937, HCC1806, HCC1935, HCC1954 and ZR75-1, MDA-MB231, MDA-MB468, MDA-MB436, MDA-MB453, MDA-MD231-derived LM2 (4175), MCF-7, MDA-MB361 and BT20 were obtained from ATCC, except indicated below. MDA-MD231-derived LM2 and 4T1 cells were a kind gift from Dr. Joan Massague (Sloan Kettering Institute) and Dr. Haifa Shen (Houston Methodist Research Institute) respectively.
Authentication	The cell were authenticated by ATCC service-STR profiling.
Mycoplasma contamination	All cell lines were not tested for mycoplasma contamination.
Commonly misidentified lines (See ICLAC register)	N/A

Animals and other organisms

Policy information about studies involving animals; ARRIVE guidelines recommended for reporting animal research

Laboratory animals	NSG (JAX stock #005557) mice were purchased from the Jackson Laboratory. SCID C. B -17 mice or BALB/c nu/nu athymic mice were purchased from Envigo.
Wild animals	N/A
Field-collected samples	N/A
Ethics oversight	The animal procedures were approved by the Institutional Animal Care and Use Committee (IACUC) of University of California, Davis.

Note that full information on the approval of the study protocol must also be provided in the manuscript.

ChIP-seq

Data deposition

☒ Confirm that both raw and final processed data have been deposited in a public database such as GEO.

☒ Confirm that you have deposited or provided access to graph files (e.g. BED files) for the called peaks.

Data access links<br>*May remain private before publication.*	https://www.ncbi.nlm.nih.gov/geo/query/acc.cgi?acc=GSE126380
Files in database submission	IP_H3K27ac_Vehicle_fastq.gz<br>IP_H3K27ac_XY018_fastq.gz<br>Input_H3K27ac_Vehicle_fastq.gz<br>Input_H3K27ac_XY018_fastq.gz<br>IP_SREBP2_Vehicle_fastq.gz<br>IP_SREBP2_XY018_fastq.gz<br>Input_SREBP2_Vehicle_fastq.gz<br>Input_SREBP2_XY018_fastq.gz<br>IP_RORgamma_Vehicle_fastq.gz<br>IP_RORgamma_XY018_fastq.gz<br>Input_RORgamma_Vehicle_fastq.gz<br>Input_RORgamma_XY018_fastq.gz<br>IP_H3K27ac_Vehicle_bw<br>IP_H3K27ac_XY018_bw<br>IP_SREBP2_Vehicle_bw<br>IP_SREBP2_XY018_bw<br>IP_RORgamma_Vehicle_bw<br>IP_RORgamma_XY018_bw<br>IP_H3K27ac_Vehicle_Peakcall_bed<br>IP_H3K27ac_XY018_Peakcall_bed<br>IP_SREBP2_Vehicle_Peakcall_bed<br>IP_SREBP2_XY018_Peakcall_bed<br>IP_RORgamma_Vehicle_Peakcall_bed<br>IP_RORgamma_XY018_Peakcall_bed
Genome browser session<br>(e.g. UCSC)	IGV

Methodology

Replicates	Two biological replicated ChIP-ed DNA were pooled for library and sequence.
Sequencing depth	We targeted on 20M clean reads for narrow-peak of H2K27ac, SREBP2 and RORgamma.
Antibodies	SREBP2 (Cayman, #10007663); H3(acetyl K27) (Abcam; ab4729) and anti-RORγ rabbit serum was generated by Covance, using purified GST-human RORγ fragment (amino acids 79-301) expressed in Escherichia coli. The specificity data for anti-RORγ antibody is shown in the Source Data. The specificity of anti-SREBP2 antibody we used has been validated by ENCODE consortium which can be accessed at the website (https://www.encodeproject.org/antibodies/ENCAB000ALD/).
Peak calling parameters	Uniquely mapped tags filtering and deduping were used for peak calling by model-based analysis for ChIP-Seq (MACS; 2.1.0) to identify regions of ChIP-seq enrichment over background. Normalized genome-wide signal-coverage tracks from raw-read alignment files were built by MACS2, UCSC tools. Effective genome size = 2.70e+09; band width = 300; model fold = [5, 50]; p-value cutoff = 1.00e-02;MACS will save fragment pileup signal per million reads.
Data quality	PBC1 (PCR Bottleneck coefficient 1)= 0.915995 (RORgamma); 0.910941(H3K27ac); 0.968991 (SREBP2). Total reads = 23615089 (RORgamma); 26140293(H3K27ac); 26229526 (SREBP2). Mapped rate % = 89.35(RORgamma); 96.52 (H3K27ac);

	85 (SREBP2). Peaks = 87346 (RORgamma);105372(H3K27ac); 69736 (SREBP2). NSC = 1.349224 (RORgamma);1.849749(H3K27ac); 1.100458 (SREBP2). RSC = 1.213267 (RORgamma); 1.489768(H3K27ac); 1.224267 (SREBP2).
Software	Bowtie2 version 2.3.4.1 for alignment MACS v2.1.0 for peak calling Homer v4.8 for enrichment UCSC tools bedClip, bed-Graph-ToBigWig for generating the bw files.
