# Supplementary material for: RORγ is a targetable master regulator of cholesterol biosynthesis in a cancer subtype
Source: Nat Commun. 2019 Oct 11;10:4621. doi: 10.1038/s41467-019-12529-3 (PMC6789042; doi:10.1038/s41467-019-12529-3)

Fig.3

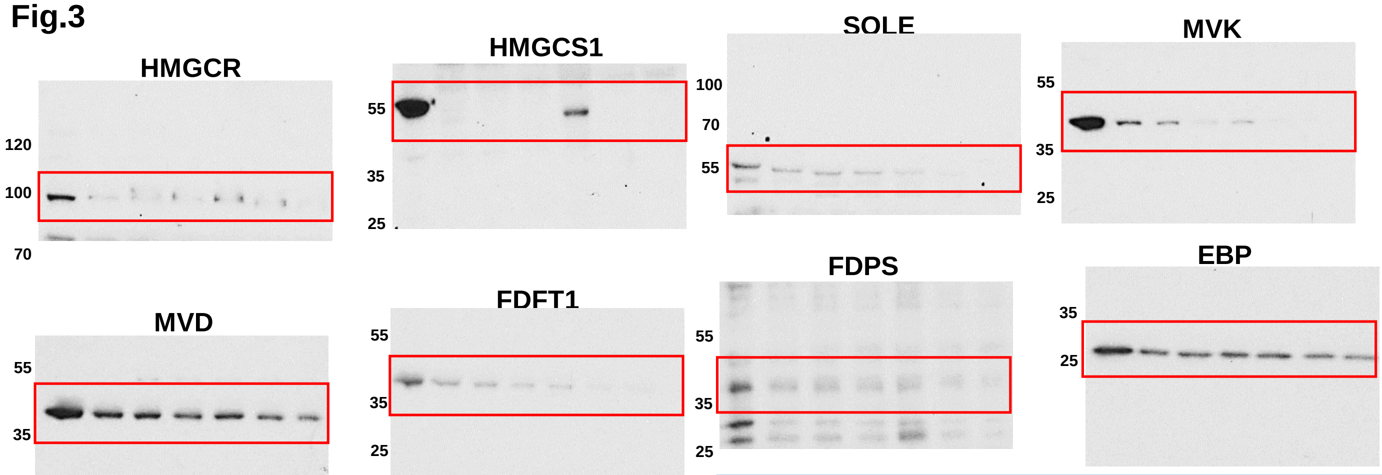

Fig.4

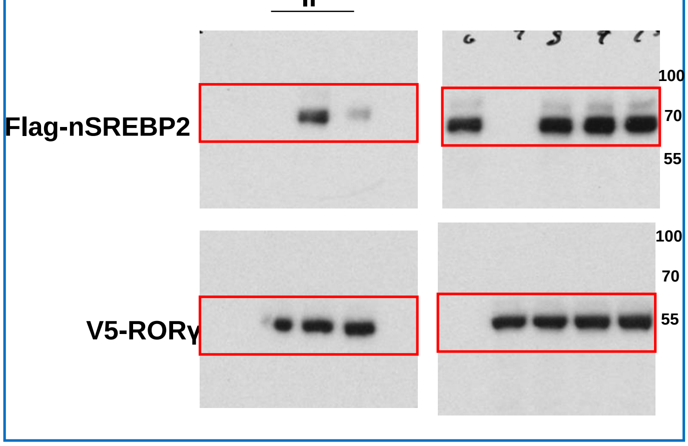

Fig.5

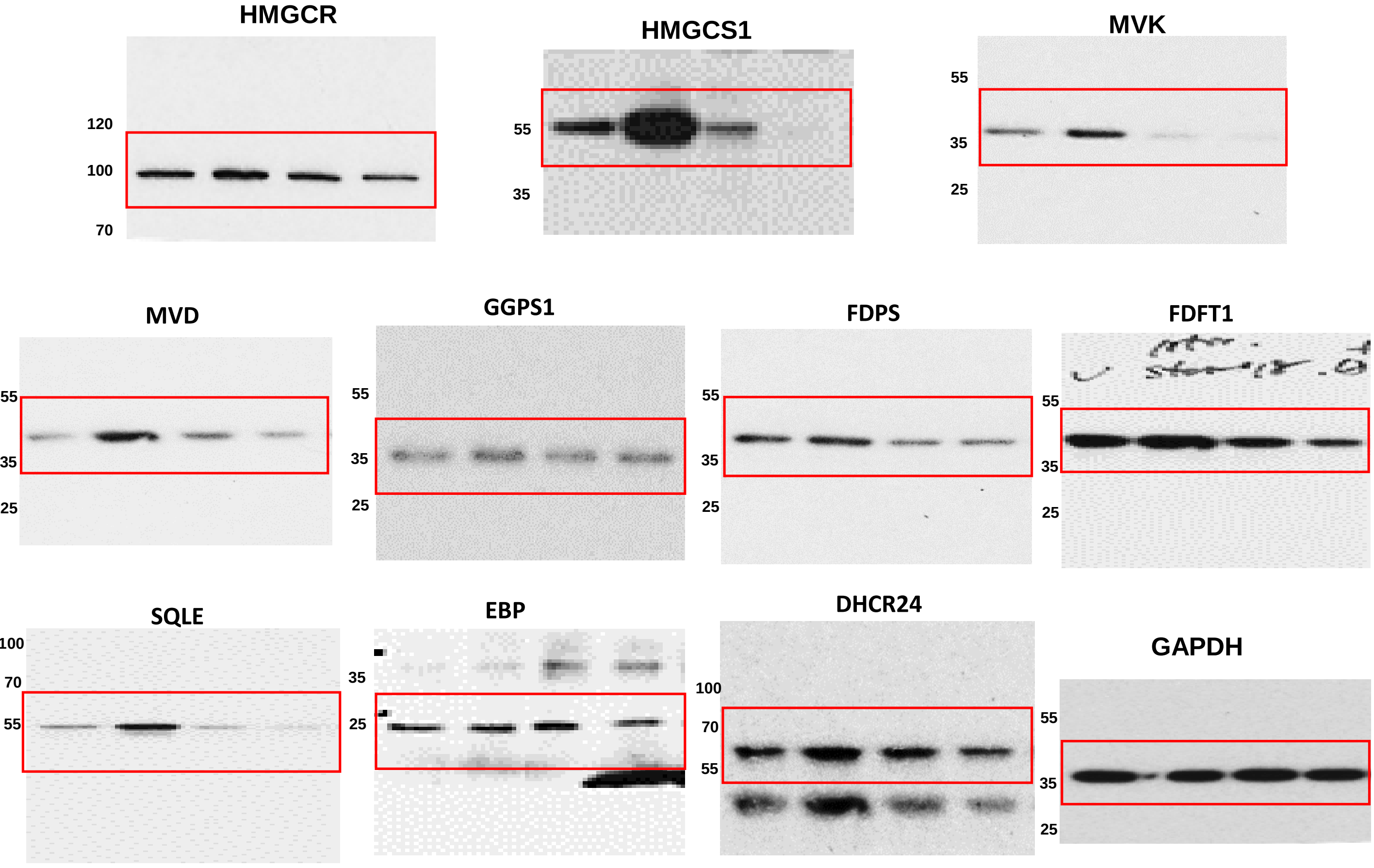

Supplementary Fig.2

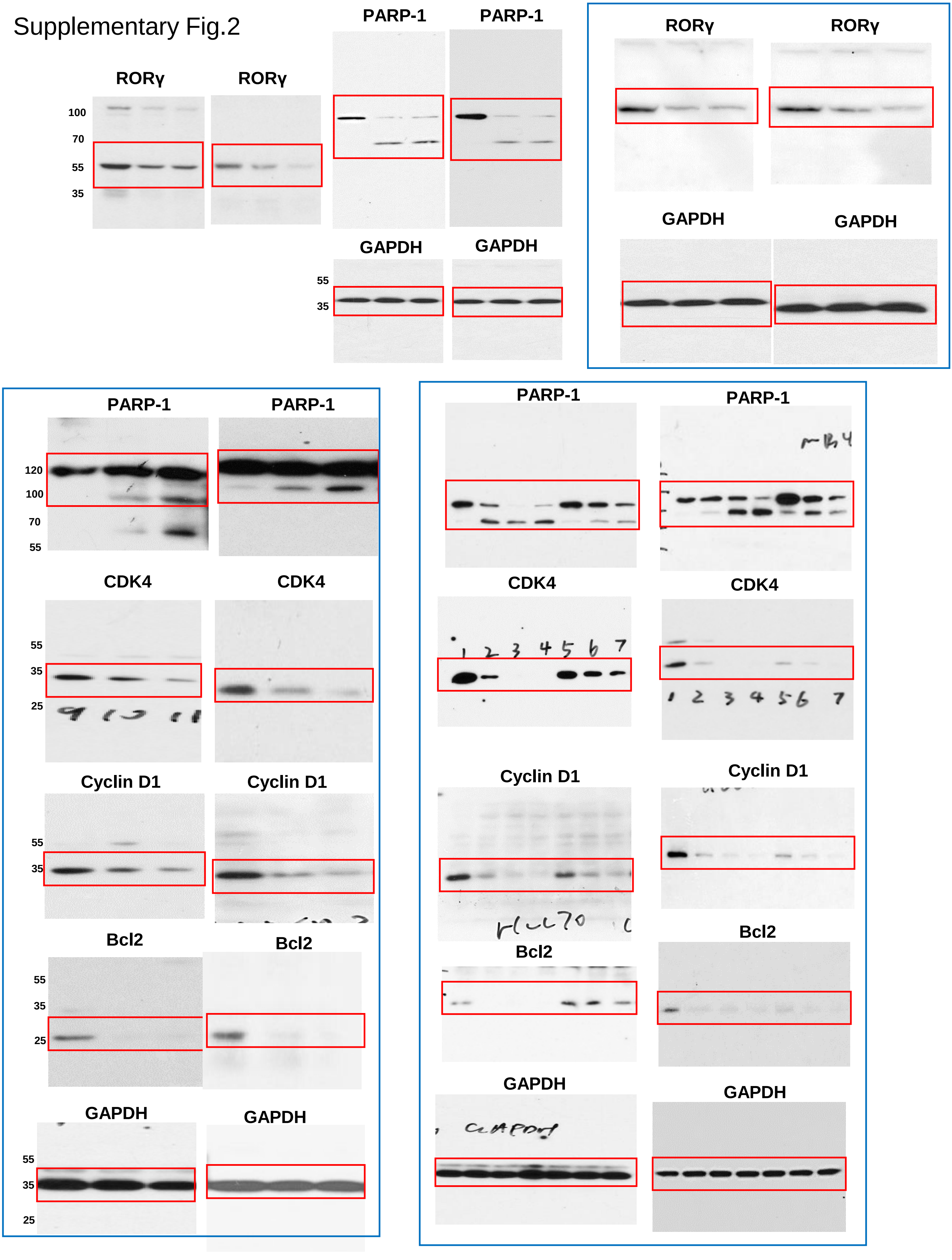

Supplementary Fig.2

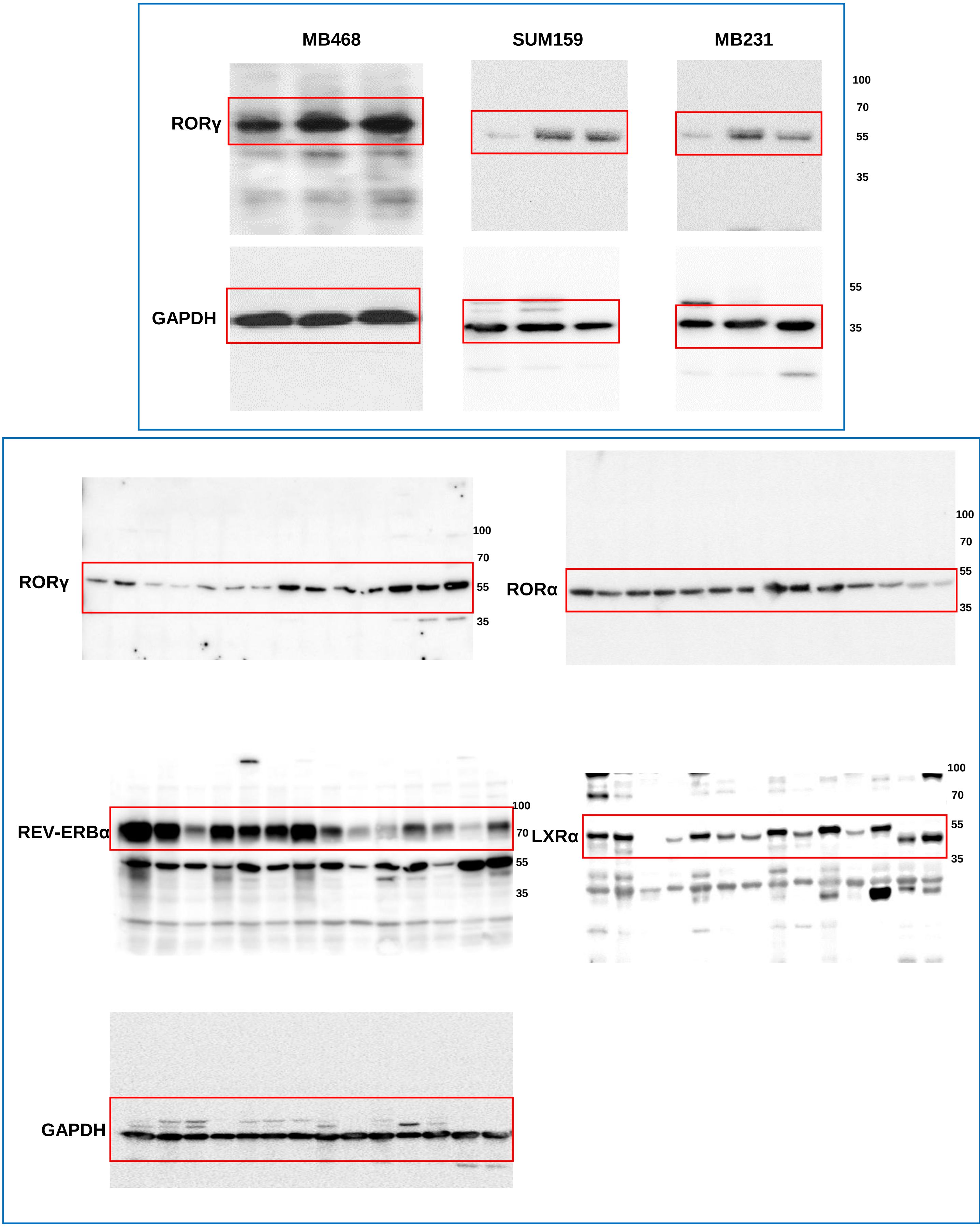

Supplementary Fig.3

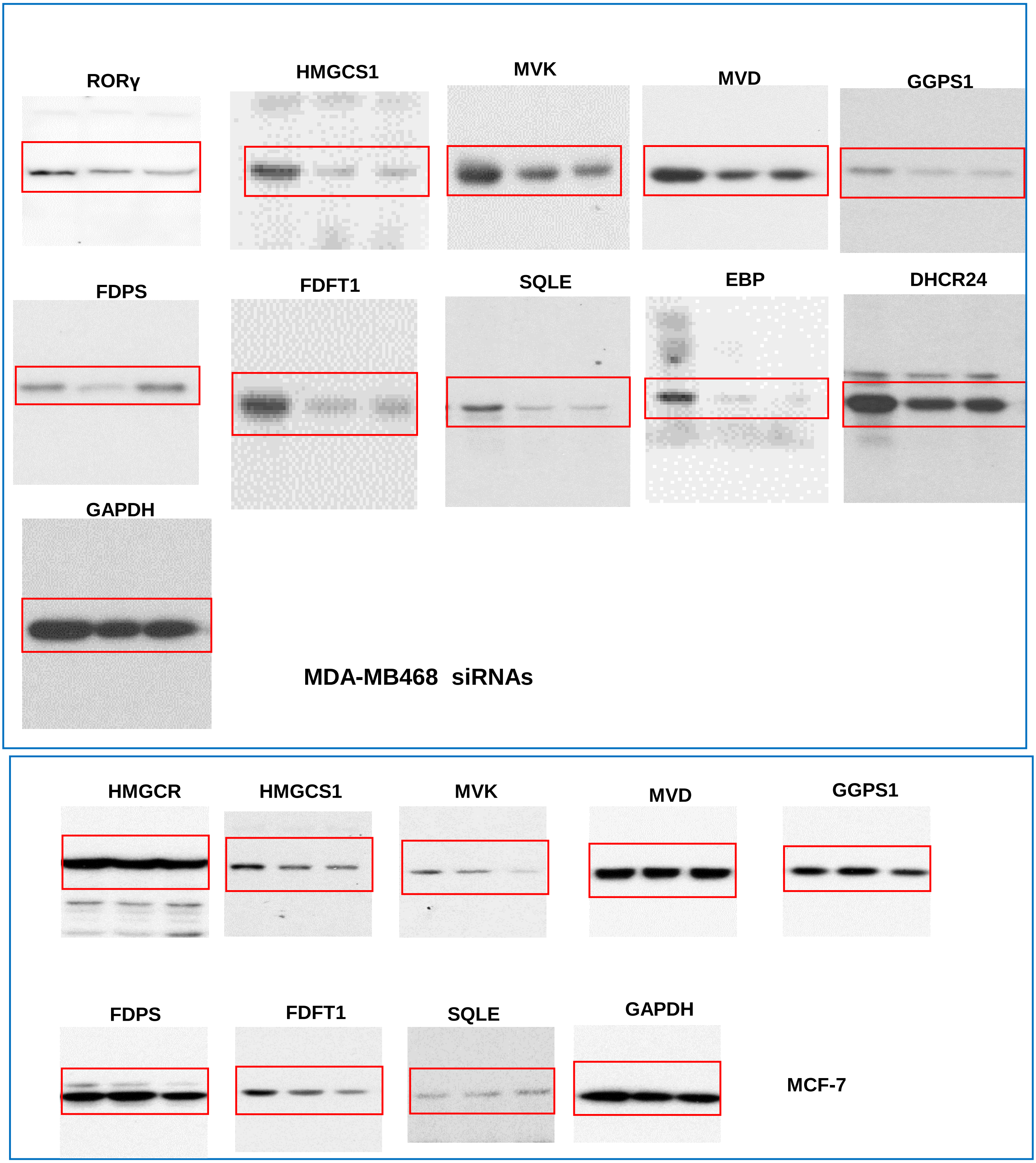

Supplementary Fig.4

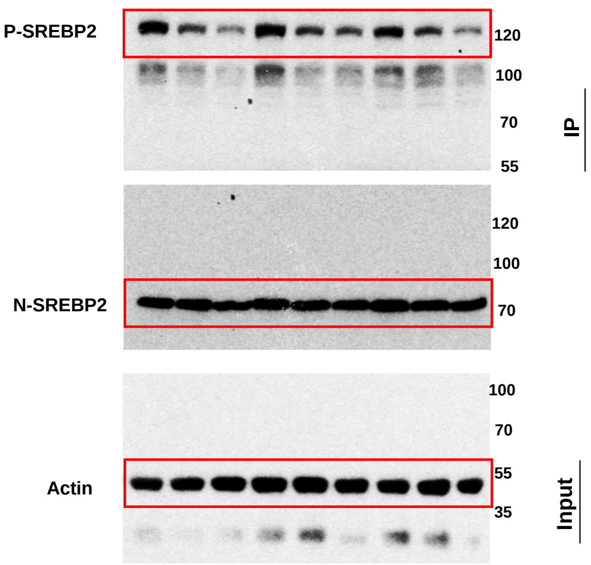

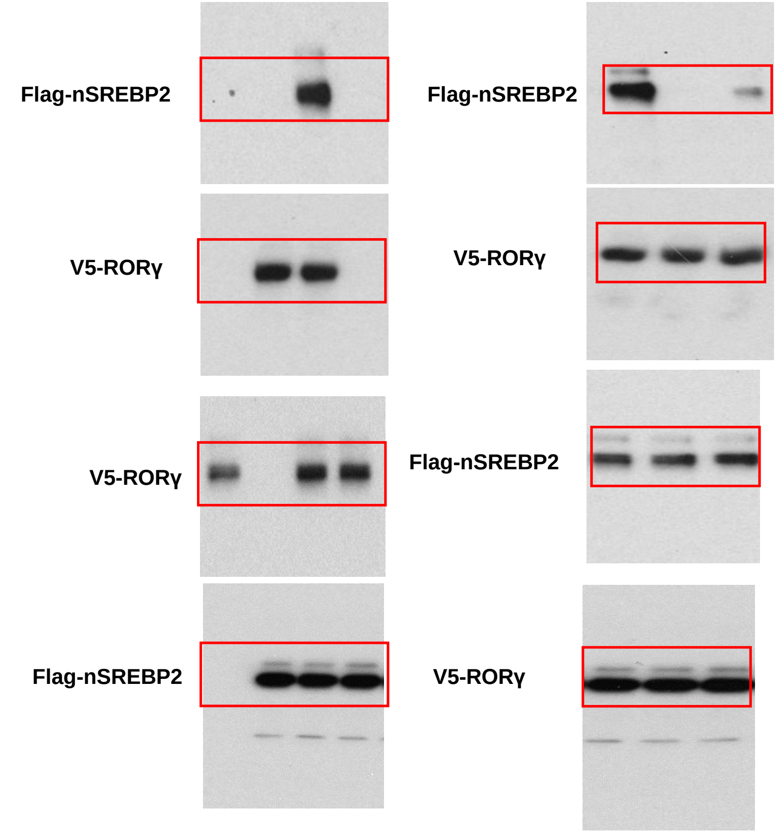

Supplementary Fig.5

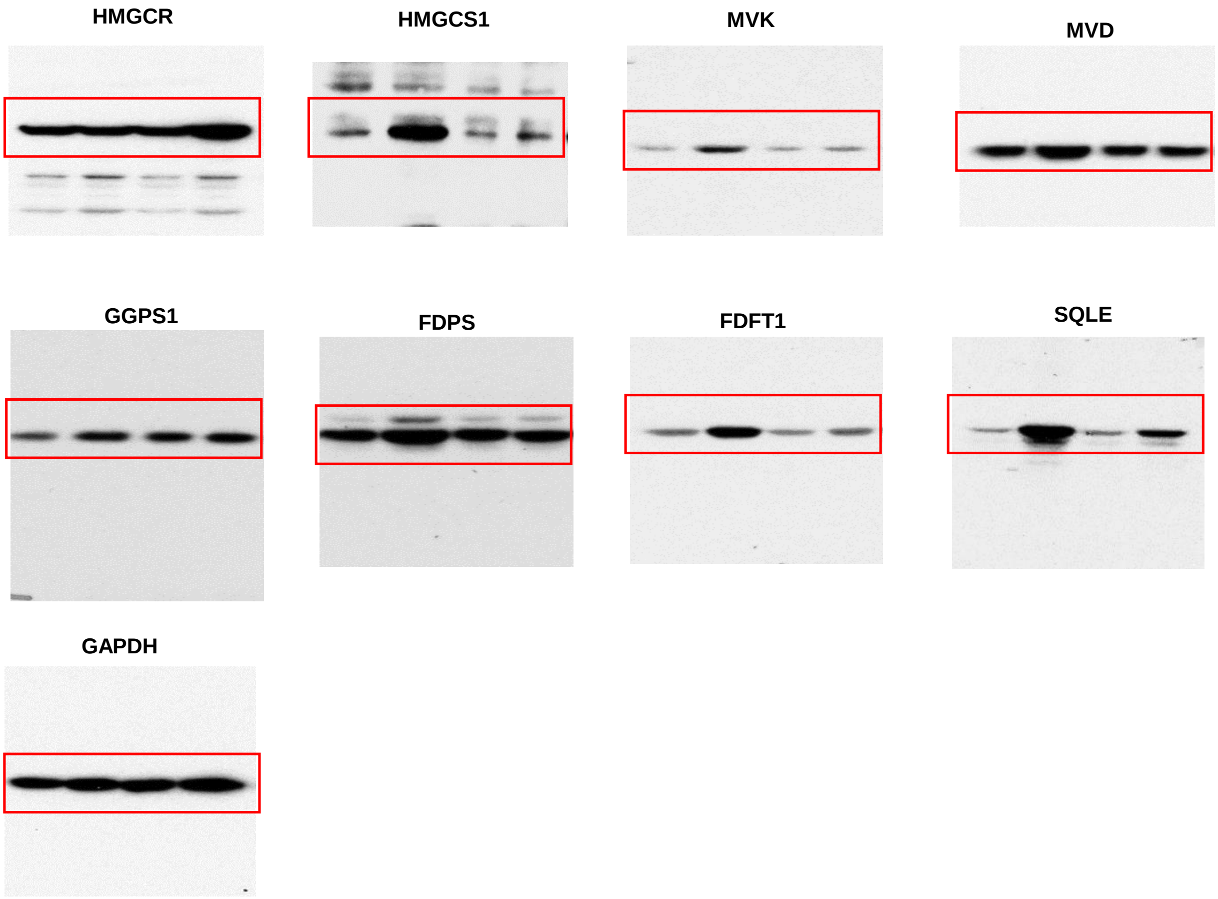

Supplementary Fig.7

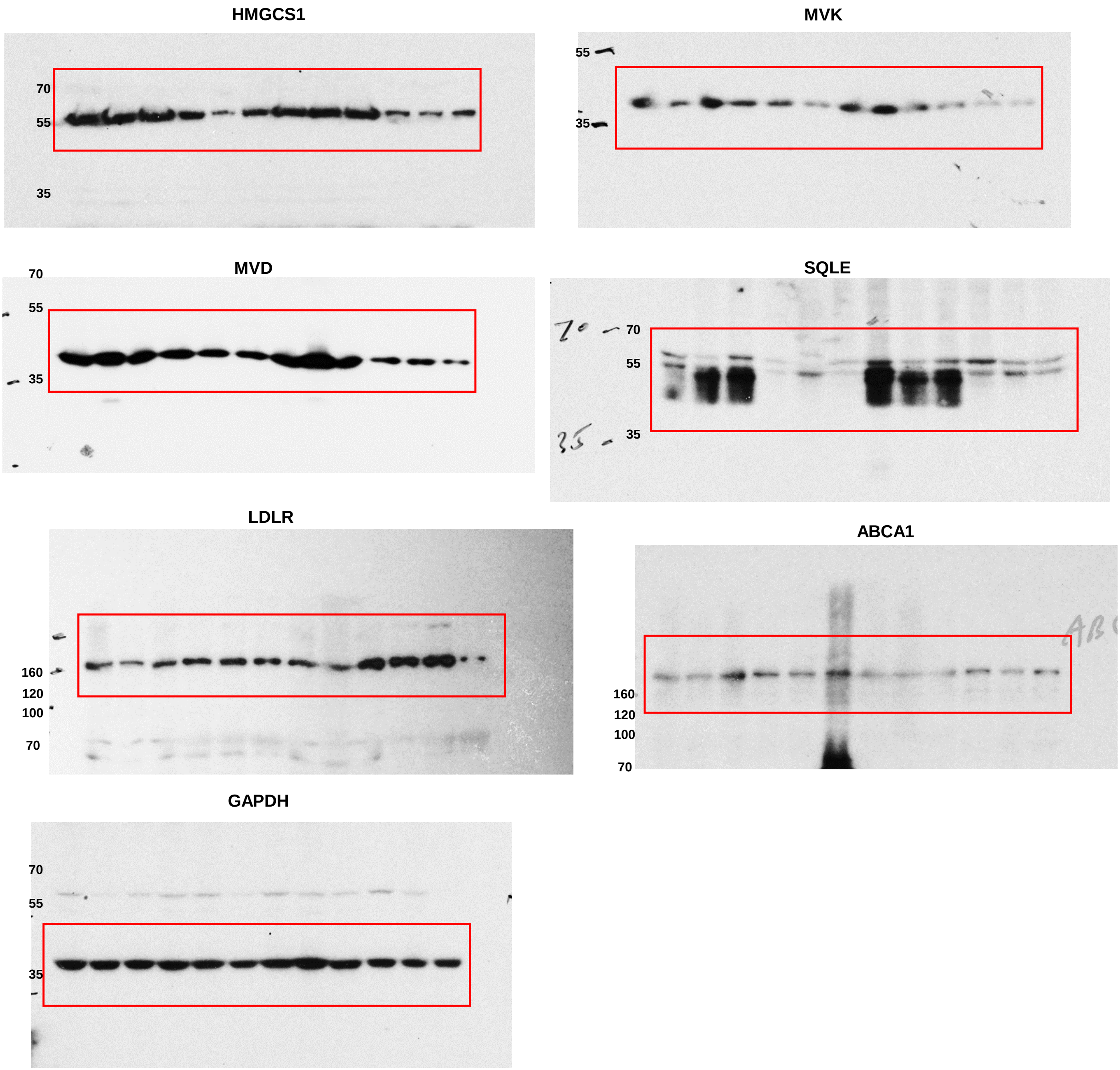

RORγ antibody validation for ChIP

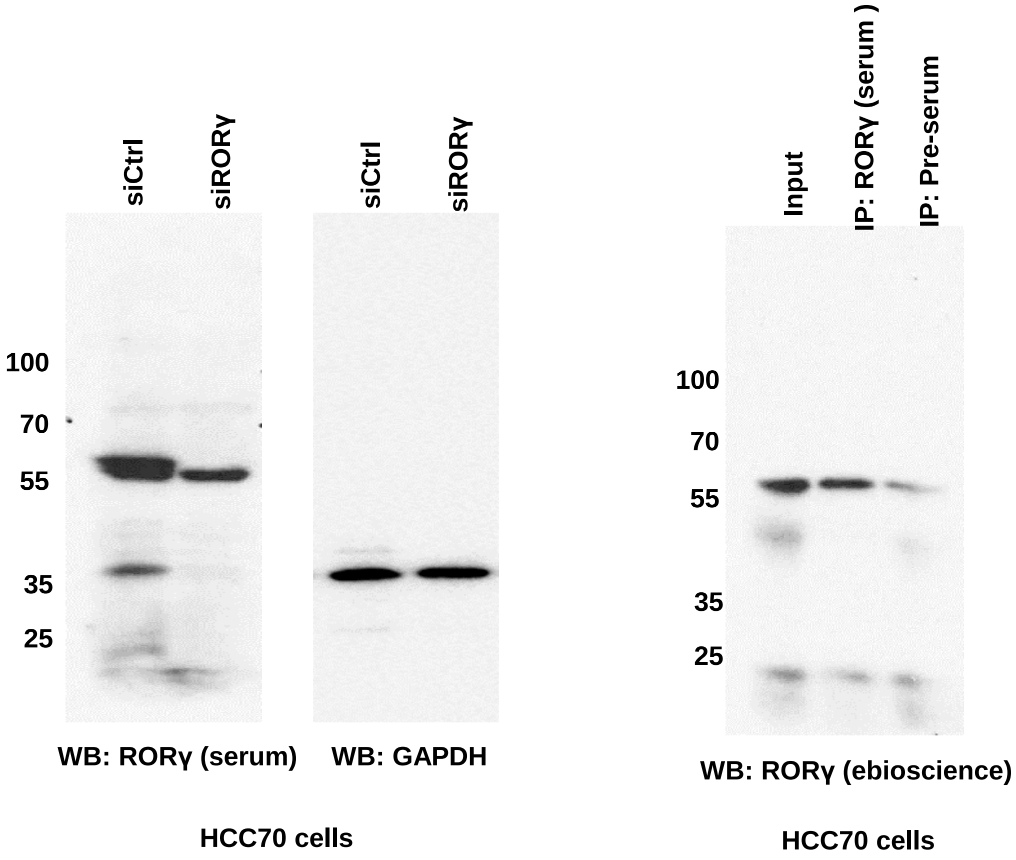

Supplement: Supplementary file 4 — Source Data [file 41467_2019_12529_MOESM4_ESM.pdf]
